# Supplementary figures and images for: The plant mobile domain proteins MAIN and MAIL1 interact with the phosphatase PP7L to regulate gene expression and silence transposable elements in Arabidopsis thaliana
Source: PLoS Genet. 2020 Apr 14;16(4):e1008324. doi: 10.1371/journal.pgen.1008324 (PMC7156037; doi:10.1371/journal.pgen.1008324)

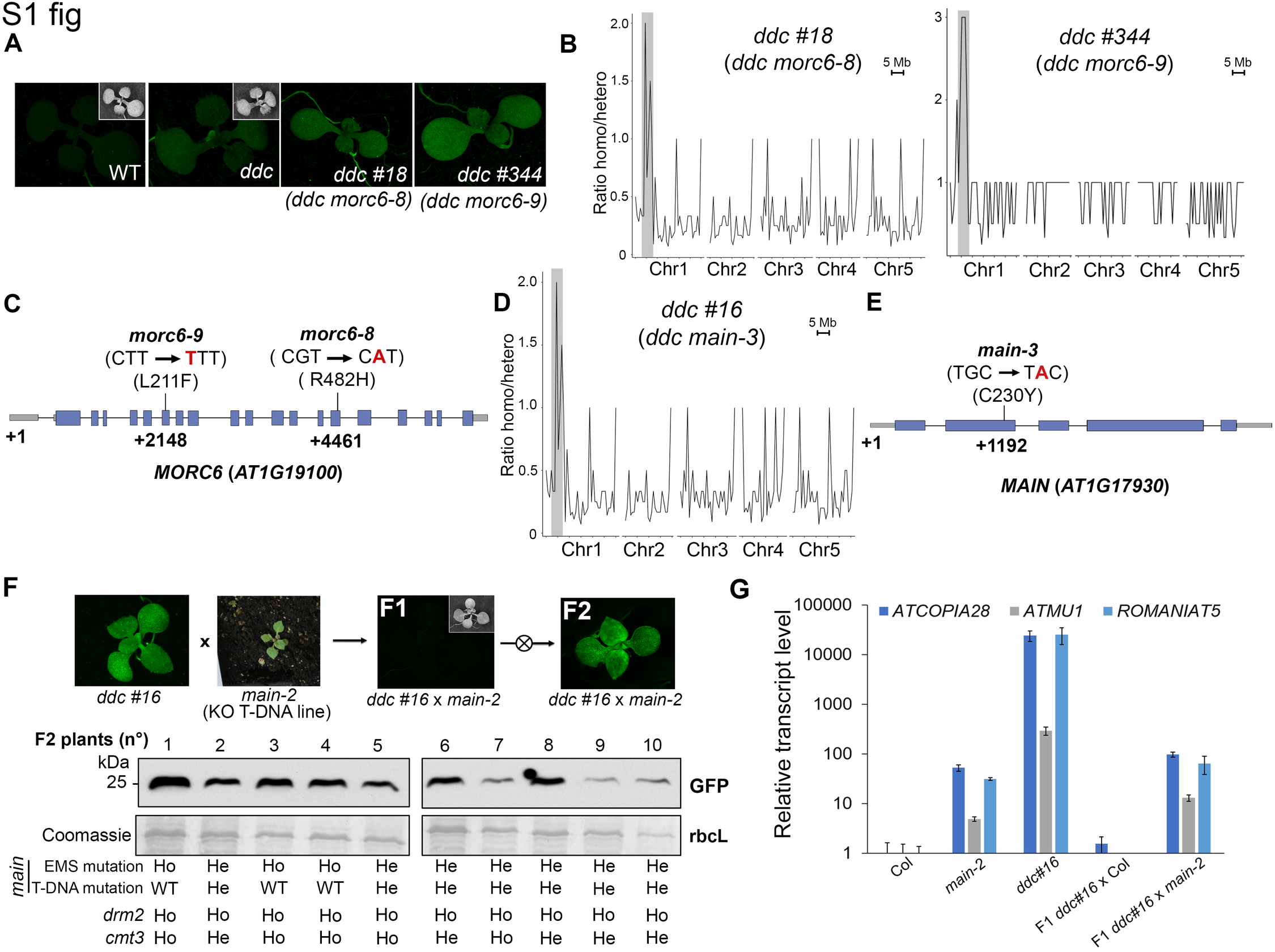

Supplement: S1 Fig — (A) Representative pictures of ddc #18 (ddc morc6-8) and ddc #344 (ddc morc6-9) mutants in comparison to ATCOPIA28::GFP WT and ddc control plants under UV light. Insets show plants under white light. (B) Enrichment in homozygote/heterozygote ratio of EMS over WT single nucleotide polymorphisms (SNPs), defining the linkage intervals for the populations ddc #18 and ddc #344. Mb: megabase. Gray-shaded rectangles delimit the mapping intervals. (C) Location of the point mutations corresponding to the morc6-8 and morc6-9 alleles within the MORC6 genomic sequence. Nucleotide and corresponding amino acid changes are indicated above the gene. Positions of the mutations are indicated relative to the transcription start site (+1). Grey boxes represent 5’ and 3’ UTR, blue boxes and lines represent exons and introns, respectively. (D) Enrichment in homozygote/heterozygote ratio of EMS over WT single nucleotide polymorphisms (SNPs), defining the linkage intervals for the population ddc #16. Gray-shaded rectangle delimits the mapping interval. (E) Location of the point mutation corresponding to the main-3 mutant allele within the MAIN genomic sequence. (F) Genetic complementation analyses using the KO T-DNA insertion line main-2. ddc #16 plants were crossed with main-2 plants. F1 plants were self-crossed, and F2 plants were screened under UV light to select GFP-overexpressing plants. Western blotting using anti-GFP antibodies confirmed GFP overexpression in selected F2 plants. Coomassie staining of the large Rubisco subunit (rbcL) is used as a loading control. KDa: kilodalton. Among the selected F2 plants, the presence of main-3 EMS and main-2 T-DNA mutant alleles were determined by dCAPS-PCR and PCR analyses, respectively. DRM2 and CMT3 genotyping were determined by PCR analyses. WT: Wild type, Ho: Homozygote mutant. He: Heterozygote. (G) Relative expression analyses of several TEs in the indicated genotypes assayed by RT-qPCR. RT-qPCR analyses were normalized using the housekeep [file pgen.1008324.s001.tif]

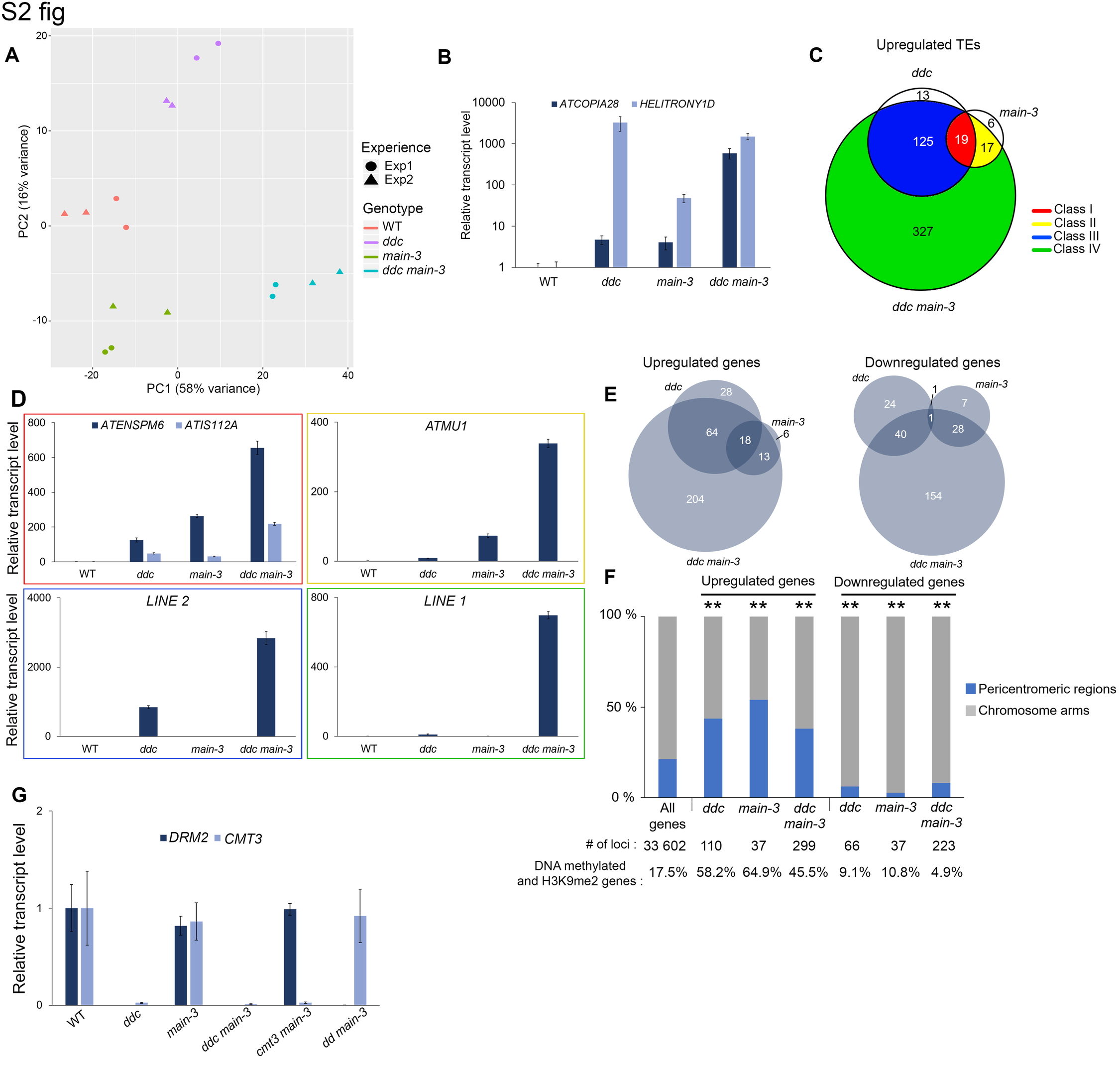

Supplement: S2 Fig — (A) Principal component analysis (PCA) performed after batch correction for first two components of the sixteen samples described in RNA-seq EMS Exp1 and Exp2. (B) Relative expression analyses of ATCOPIA28 and HELITRONY1D (AT5TE35950) in ddc, main-3 and ddc main-3 assayed by RT-qPCR. RT-qPCR analyses were normalized using the housekeeping RHIP1 gene, and transcript levels in the different genotypes are represented relative to WT. Error bars indicate standard deviation based on three independent biological replicates. (C) Venn diagrams analysis showing the overlaps between reproducibly upregulated TEs in ddc, main-3 and ddc main-3. Fisher's exact test statistically confirmed the significance of Venn diagram overlaps (p-value <2.2.10e-16). (D) Same as panel B for TEs defined as class I-IV TEs. Frames of RT-qPCR graphs are using the same color code as shown in panel C. (E) Venn diagrams analyses defining the overlaps between up- and downregulated genes in the different genotypes. Fisher's exact test statistically confirmed the significance of Venn diagram overlaps (p-value <2.2.10e-16). (F) Fraction of misregulated genes in ddc, main-3 and ddc main-3 located in chromosome arms or in pericentromeric regions as defined in [50]. Asterisks indicate statistically significant enrichments of misregulated genes in chromosome arms or pericentromeric regions in comparison to the genomic distributions of all A. thaliana genes (Chi-Square test, **: p-value≤ 0.01). Percentages of genes targeted by DNA methylation and H3K9me2 were calculated based on enrichment in heterochromatin states 8 and 9 as defined in [51]. (G) Relative expression analyses of DRM2 and CMT3 in ddc, main-3, ddc main-3, cmt3 main-3 and dd main-3 assayed by RT-qPCR. RT-qPCR analyses were normalized using the housekeeping RHIP1 gene, and transcript levels in the different genotypes are represented relative to WT. Error bars indicate standard deviation based on three independent biological replicates. Screening of [file pgen.1008324.s002.tif]

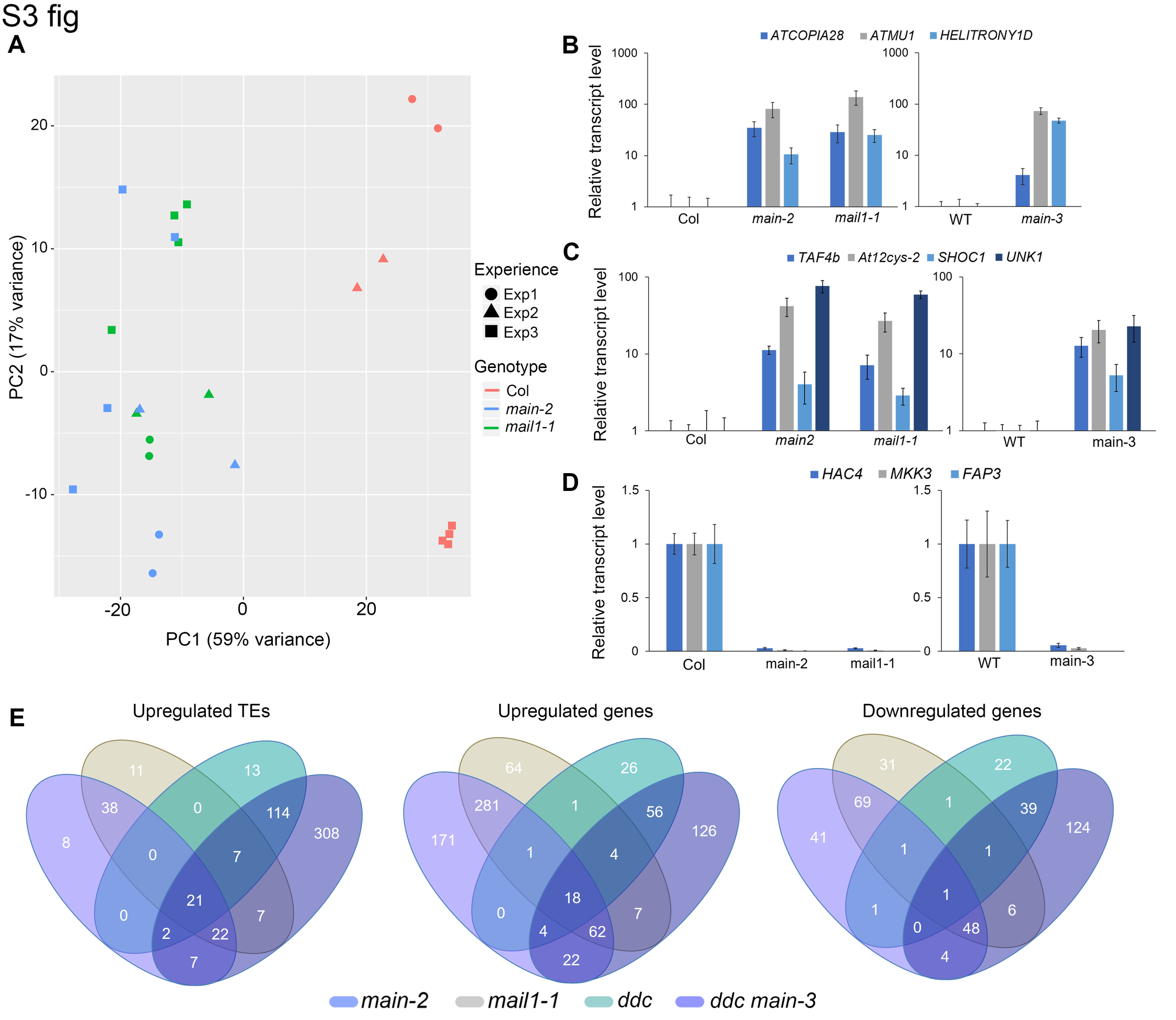

Supplement: S3 Fig — (A) Principal component analysis (PCA) performed after batch correction for first two components of the twenty-four main-2, mail1-1 and WT Col samples described in RNA-seq Exp1, Exp2 and Exp3. (B-D) Relative expression analyses of several upregulated TEs (B), upregulated genes (C), and downregulated genes (D) in main-2, mail1-1 and main-3 assayed by RT-qPCR. RT-qPCR analyses were normalized using the housekeeping RHIP1 gene, and transcript levels in the different genotypes are represented relative to respective WT controls. Error bars indicate standard deviation based on three independent biological replicates. (E) Venn diagrams analyses representing the overlaps between misregulated loci in main-2, mail1-1, ddc and ddc main-3. Fisher's exact test statistically confirmed the significance of Venn diagram overlaps (p-value <0.005). (TIF) [file pgen.1008324.s003.tif]

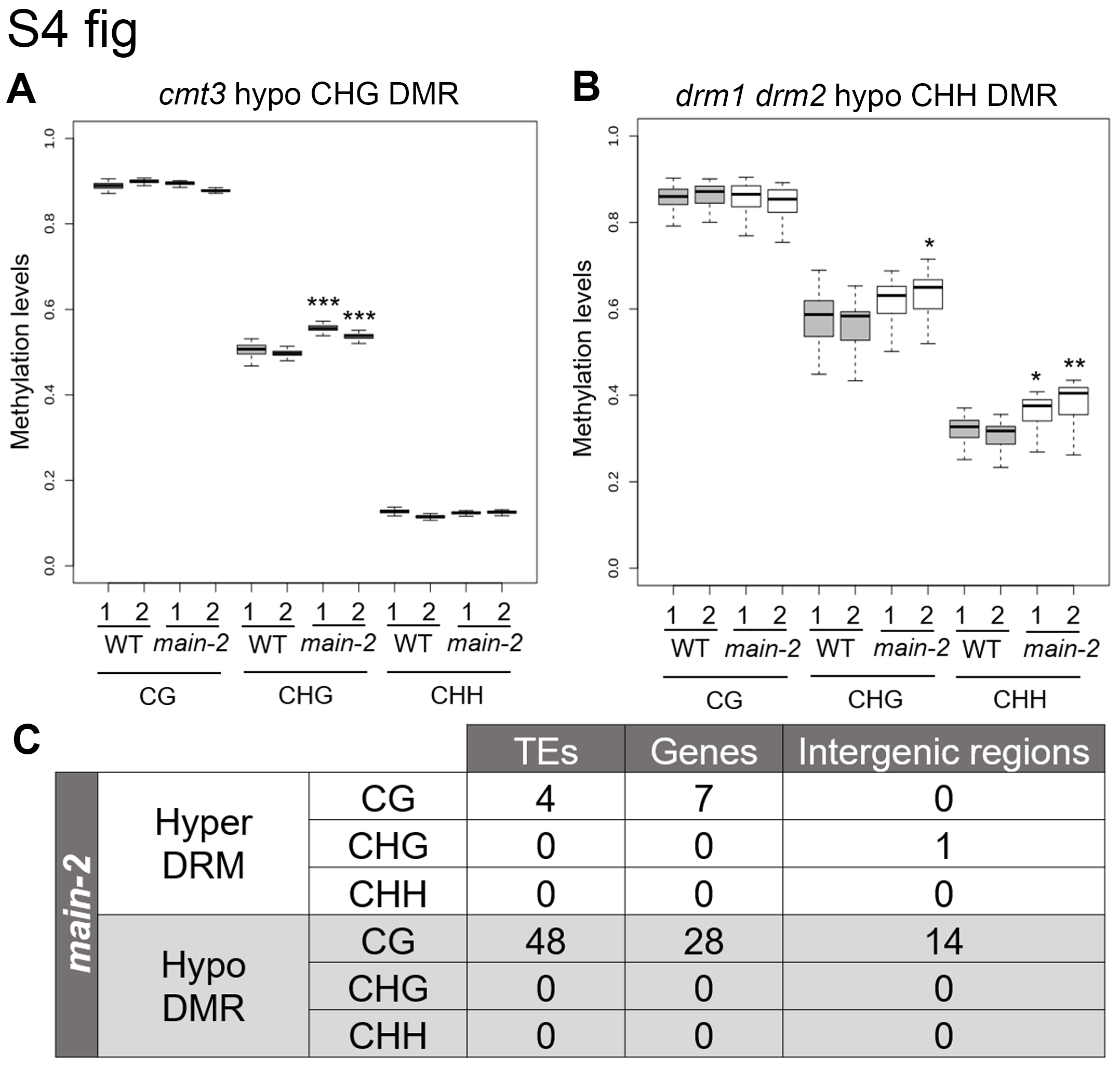

Supplement: S4 Fig — (A-B) Boxplot analyses in two main-2 and WT Col biological replicates showing the DNA methylation levels at genomic sites previously defined as hypo CHG differentially methylated regions (DMR) in cmt3 (A) and hypo CHH DMR in drm1 drm2 (B) based on [26]. p-values were calculated using a Wilcoxon test. *: p-value <5.10e-7, **: p-value <5.10e-10, ***: p-value < 2.10e-16. (TIF) [file pgen.1008324.s004.tif]

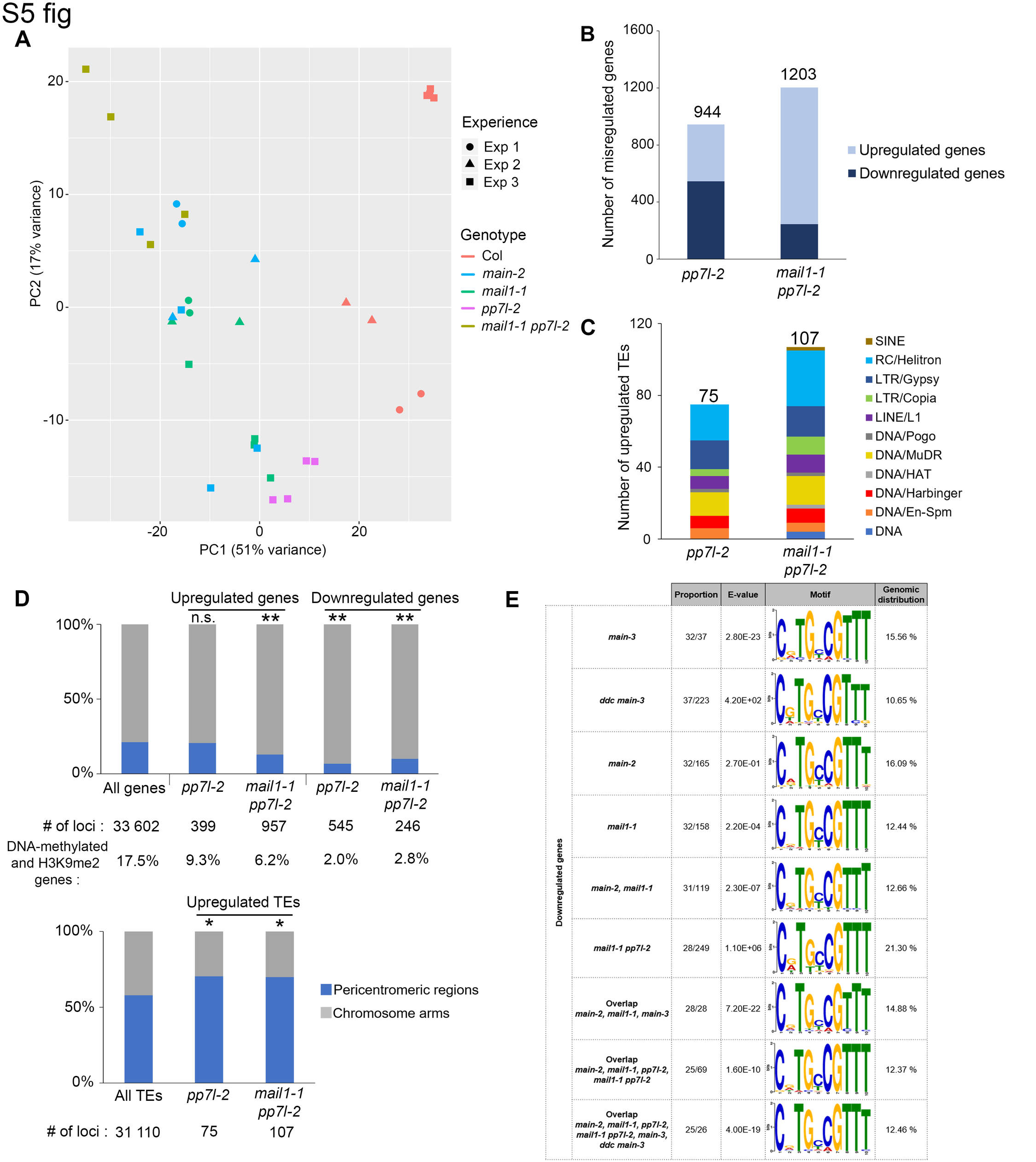

Supplement: S5 Fig — (A) Principal component analysis (PCA) performed after batch correction for first two components of the thirty-two samples described in RNA-seq Exp1, Exp2 and Exp3. (B) Number of misregulated genes in the different genotypes in comparison to WT Col plants from RNA-seq Exp3 (four biological replicates, S3 and S6 Tables). (C) Number of upregulated TEs in pp7l-2 and mail1-1 pp7l-2, and classified by TE superfamily. (D) Fraction of misregulated loci in pp7l-2 and mail1-1 pp7l-2 located in chromosome arms or in pericentromeric regions as defined in [50]. Asterisks indicate statistically significant enrichments of downregulated genes, upregulated genes and TEs in chromosome arms and pericentromeric regions, respectively, in comparison to the genomic distributions of all A. thaliana genes and TEs (Chi-Square test, *: p-value≤ 0.05, **: p-value≤ 0.01, n.s: not significant). Percentages of genes targeted by DNA methylation and H3K9me2 were calculated based on enrichment in heterochromatin states 8 and 9 as defined in [51]. (E) Identification and proportions of the ‘DOWN’ DNA motif among the promoters of downregulated genes and all Arabidopsis genes using the MEME software. Promoter regions are defined as 1kb upstream of ATG. The list of all Arabidopsis genes used to determine genomic distributions is based on the TAIR file: TAIR10_upstream_1000_translation_start_20101028. RNA-seq threshold: log2≥2, or log2≤-2; p-adj< 0.01. (TIF) [file pgen.1008324.s005.tif]

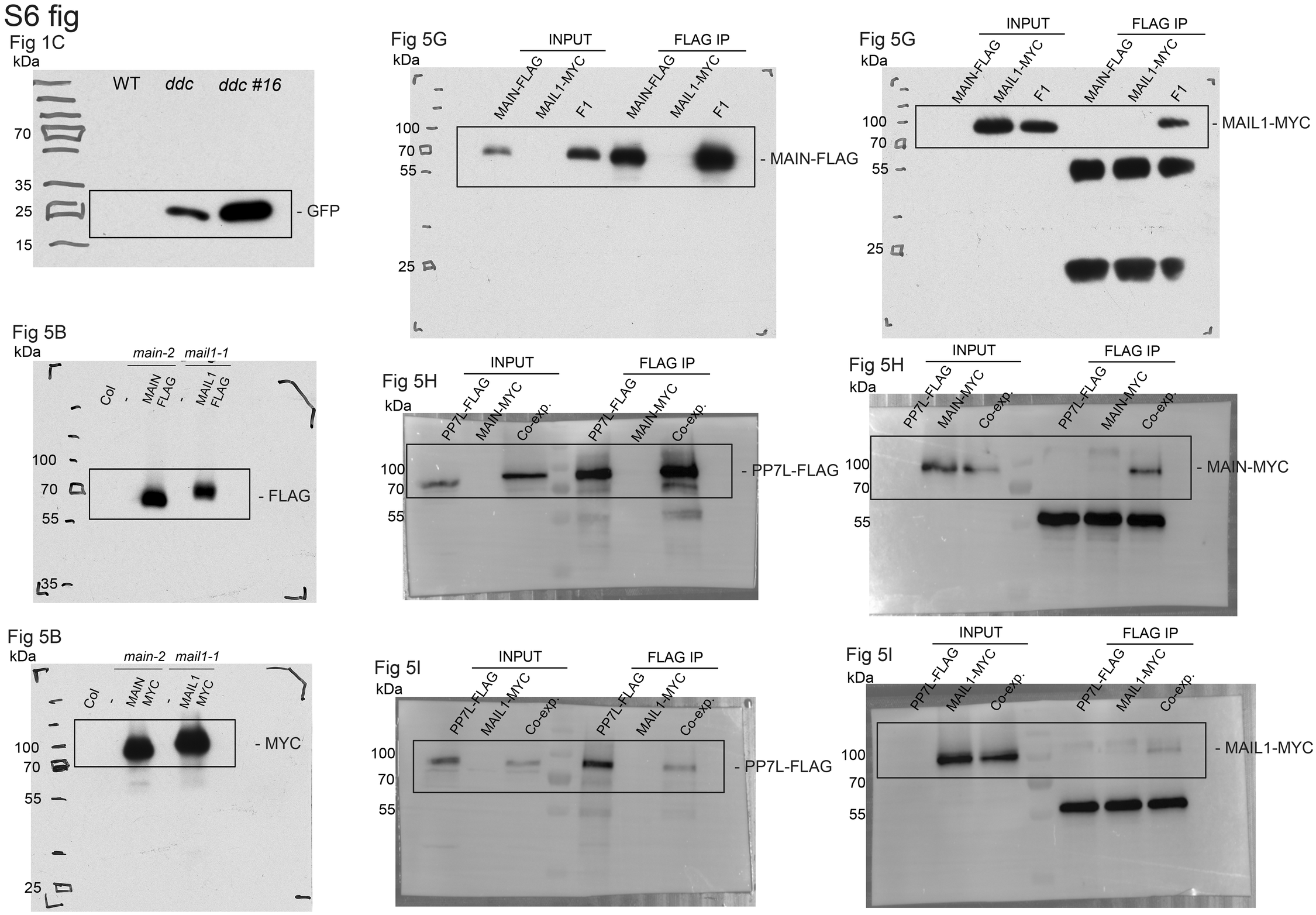

Supplement: S6 Fig — (TIF) [file pgen.1008324.s006.tif]
