# Supplementary material for: The plant mobile domain proteins MAIN and MAIL1 interact with the phosphatase PP7L to regulate gene expression and silence transposable elements in Arabidopsis thaliana
Source: PLoS Genet. 2020 Apr 14;16(4):e1008324. doi: 10.1371/journal.pgen.1008324 (PMC7156037; doi:10.1371/journal.pgen.1008324)
Supplement: Attachment — Submitted filename: Nicolau et al Main Mail1.docx [file pgen.1008324.s020.docx]

In the current submission, Nicolau and colleagues combine genetic, genomic, and phylogenetic approaches to provide new insights into the roles of two plant mobile domains (PMD) proteins, MAIN and MAIL1, in regulating the expression of both transposable elements (TE) and protein coding genes. Previously, mutants in these two gene were shown to be required for TE silencing, genome stability and plant development. However, it remained unclear how these factors fit into other known transposon silencing pathways and how, mechanistically, they control gene expression and developmental processes. Here, the authors conducted an EMS-based enhancer screen in a drm1,drm2,cmt3 (ddc) background already deficient in 2 of the 3 main DNA methylation pathways and identified two new alleles of *morc6* (a previously characterized silencing factor that acts downstream of DNA methylation) and one new allele of *main*. On a global scale they were able to show that the *ddc main-3* quadruple mutant has a much stronger phenotype than the *ddc* or *main-3* mutants alone, demonstrating that these factors likely act in independent pathways that together are required to repress TEs, especially those located in pericentromeric heterochromatin. To gain mechanistic insight into how MAIN1 functions, the authors affinity purified both MAIN and the related MAIL1 proteins using complementing, epitope tagged, transgenic plant lines and identified PP7L as a co-purifying factor by Mass Spectrometry. After confirming these interactions by as series of co-IP experiments, the authors then focused on determining whether *pp7l* mutants showed similar phenotypes as observed of *main* and *mail1* mutants. Indeed, *pp7l* mutants show similar growth defects (i.e. they are developmentally delayed) and by mining of publicly available transcriptomic data they found that although *pp7l* mutants show stronger gene expression defects, a significant subset of upregulated TEs and mis-regulated genes are shared with the *main* and *mail1* mutants. Further investigation into these common genic targets revealed an over-represented motif, suggesting this complex (either directly or indirectly) regulates gene expression via recognition of a conserved cis-regulatory element. Finally, the authors noted that while MAIN and MAIL1 associated with a PPP domain protein (*i.e.* PP7L) other PMD proteins themselves already contain PPP domains. To better understand the relationships between these domains, the authors conducted phylogenetic analyses that identified two independent case for the association and neofunctionalization of PMD-C and PP7 domains. Taken together these studies demonstrate that MAIN forms a stable complex with MAIL1 and PP7 and that all these factors influence the expression of both genes and TEs, some of which are associate with a shared cis-regulatory motif. In addition, they show that the relationship between PMD and PPP domains is conserved, suggesting this combination of motifs might represent a new protein module important for gene regulation. Although some clarifications/analyses are needed (see below), the data are well controlled, support the authors main conclusions, and advance our understanding of how the MAIN complex influences gene expression.

Comments

1. In several places the authors mention MAIN acting synergistically and redundantly with DNA methylation to silence TEs. This is confusing as written since it is not possible for MAIN to be both at any given site. Rather, MAIN is either synergistic or redundant depending on the locus. The authors should clarify this throughout.
2. On page 6, line140-143: For the text introducing this paragraph it is hard to determine what is new data and what is referring to previously published findings. Please clarify.
3. For the qPCR in Fig.1C/F it is not completely clear how the data is being normalized. From the legend it seems it might be normalized to both the WT control and a house keeping gene?
A. Please clarify the normalization procedure and if it is double normalized please clarify why this is required as it could affect the data interpretation especially if the genes/TEs being assayed are very lowly expressed in the control plants.
B. To convey the robustness of the gene expression changes for these mutants and complement the data shown in Fig. 1F, please include heatmaps showing the expression of levels of the genes in the different categories shown in Fig. 1E using the RNAseq data from these mutants. Perhaps a heatmap would be a nice format to use.
4. Is the qPCR shown in Fig 3D-F normalized differently than in Fig 1? The descriptions of the normalization in the legends differ. If they are the same, please make the descriptions similar. If they differ, please reanalyze the data in a similar manner so the data can be compared between figures.
5. It is not clear why the authors don’t include their *main-3* mRNA seq data for protein coding genes in any of the figures. The DE genes are listed in the supplemental table, but this information is not included in Fig S2C and it is not directly compared to the previously published *main-2* data. Please include this comparison. Even if the *main-3* point mutant is weaker than the null *main-2* allele, one would still expect a similar trend.
6. Please provide tables for the NGS data showing what was sequenced and the relevant mapping and coverage statistics.
7. For their complementation assays, the authors look at the expression of several TEs in Fig. S2F, but they don’t show data for any protein coding genes. Do these lines also complement at genes?
8. It would be helpful to provide more information about the connections between the roles of the MAIN/MAIL1/PP7L complex and DNA methylation. MAIN has a clear connection based on the *ddc main-3* phenotype. However, it remains unclear if MAIN has two separate roles, one in connection to DNA methylation and another in connection with MAIL1 and PP7L. Perhaps addressing some of the following questions will shed light on this aspect of MAIN function:
   a. Are there significant changes in methylation outside of the DE genes and TEs in *main* mutants?
   b. Is there methylation at the C’s within the CxTGxCGTTT motif in the promoters of the genes that are mis-regulated in the *main*, *mail1*, and/or *pp7l* mutants? If so, does the methylation at these specific sites change in the *main* mutant based on the BS-seq data?
   c. Are the DE genes and TEs in *pp7l* also enriched in pericentromeric heterochromatin? Do they overlap with any of the DE genes/TEs in the *ddc main-3* mutant?
   d. Does the *ddc pp7l* mutant phenocopy the *ddc main-3* mutant in terms of gene/TE mis-regulation?

In the absence of additional support connecting the MAIN/MAIL1/PP7L complex to DNA methylation, the possibility that MAIN serves a dual role in several different gene regulation pathways for TE vs gene regulation should be included in the discussion.

9. The phylogenetic analyses appear to be sound. However, as I am not an expert, I defer to other reviewers should issues be raised.

Minor comments:

-) In the methods (page 14, line 353) the authors mention in vitro plant culture, but this is not mentioned in the rest of the methods. Please clarify what experiment used plant cultures.

-) For Figure 2A, please address the following:

A. It is not clear from the methods that the controls were done in parallel with the MAIN and MAIL1 IPs and if they were all done in parallel, why there are only two experiments for MAIL1. Please clarify.

B. Are these the only proteins identified consistently between replicates or are these just the top hits? I was not able to access the MS data using the information provided with the submission, but regardless, such information should be mentioned, even if briefly.

-) In Fig 3 please add the allele information for each mutant and, as was done in the supplemental figure, please indicate in the legend the references for the mRNA-seq datasets analyzed for each of these mutants.

-) In Fig S1C it would be helpful to mark the relevant interval on each of the three plots.

-) In Fig S3G and Table S3, I believe the number of downregulate genes for *ddc main-3* is 87 not 88. Please fix as needed.
