# Supplementary material for: The plant mobile domain proteins MAIN and MAIL1 interact with the phosphatase PP7L to regulate gene expression and silence transposable elements in Arabidopsis thaliana
Source: PLoS Genet. 2020 Apr 14;16(4):e1008324. doi: 10.1371/journal.pgen.1008324 (PMC7156037; doi:10.1371/journal.pgen.1008324)
Supplement: Attachment — Submitted filename: Nicolau et al_Response to reviewers.docx [file pgen.1008324.s021.docx]

GENOME AND PLANT DEVELOPMENT LABORATORY – LGDP

NATIONAL SCIENTIFIC RESEARCH CENTER – CNRS

UNIVERSITY OF PERPIGNAN – UPVD

November 29^th^, 2019

Dear Claudia Köhler, Wendy Bickmore and PLOS Genetics editors,

Thank you very much for considering our manuscript entitled ‘**'The plant mobile domain proteins MAIN and MAIL1 interact with the phosphatase PP7L to regulate gene expression and silence transposable elements in Arabidopsis thaliana'** for publication in PLOS Genetics.

We have now greatly revised our manuscript, addressing most of the reviewers and editors’ comments. **The manuscript is largely improved with the addition of several main figures and supporting material.** **It is now composed of eight main figures, six supporting figures and fourteen supporting tables.**

The original figure 1 has been split into two figures. Figure 1 reports the identification of the hypomorphic *main-3* mutant allele from the EMS screen; and figure 2 describes the genetic interaction between MAIN, DRM2 and CMT3. Figures 1 and 2 are supported by S1 and S2 Figs, respectively. Following Reviewers and editors’ suggestions, we have made a new figure 3 describing the comparative analysis of misregulated loci in *main-2, mail1-1* and *main-3*; and a new figure 4 depicting statistical analyses of DNA methylation in *main-2*. Figures 3 and 4 are supported by S3 and S4 Figs, respectively. Figure 5 is now combining the complementation analyses of epitope-tagged MAIN and MAIL1 lines, together with the biochemical studies that identified PP7L as MAIN/MAIL1 interactor (originally described in S2 Fig and Fig 2, respectively). The new figure 6 describes the transcriptomic analyses of *pp7l-2* single and *mail1-1 pp7l-2* double mutants, as well as the overlaps among misregulated loci between *pmd* and *pp7l* single and double mutants. Figure 6 is supported by S5 Fig. The new Figure 7 shows subnuclear distribution of constitutive heterochromatin in *pp7l-2* based on H3K9me2 immunostaining. Finally, originally described in old figure 4, the phylogenetic analyses of PMD and PP7/PP7L domains in eudicots is now reported in figure 8. Altogether, we believe that these new figures are strongly strengthening the manuscript.

Thank you very much for considering this revised version of our manuscript. We hope you will appreciate all the modifications we have made, and that the manuscript is now acceptable for publication in PLOS Genetics.

Sincerely yours,

Guillaume Moissiard

LGDP UMR5096 CNRS/UPVD

Bâtiment T

58, Avenue P. Alduy - 66860 Perpignan FRANCE

Tel : +33(0) 4 68 08 67 90

[guillaume.moissiard@univ-perp.fr](mailto:guillaume.moissiard@univ-perp.fr)

http://lgdp.univ-perp.fr/index.php?page=pontvianne-moissiard-team

**Responses to Reviewers and Editor comments**

We thank the reviewers and editors for all the constructive and encouraging comments. Below are our point-by-point responses. **Editors and reviewers’ comments are in** **bold type**, and our responses are in regular type, blue color.

**Dear Dr Moissiard,**

**Thank you very much for submitting your Research Article entitled 'The plant mobile domain proteins MAIN and MAIL1 interact with the phosphatase PP7L to regulate gene expression and silence transposable elements in Arabidopsis thaliana.' to PLOS Genetics. Your manuscript was fully evaluated at the editorial level and by independent peer reviewers. The reviewers appreciated the attention to an important problem, but raised some substantial concerns about the current manuscript. In particular, the reviewers asked to substantiate the relationship between MAIN/MAIL1 and PP7L by adding detailed information on changes of the transcriptome and methylome and by adding information on double mutants of main/mail1 and pp7l. I agree that adding additional information would strengthen this manuscript. The reviewers made several suggestions to improve the transcriptome and methylome analyses that the authors could follow. Based on the methods I had the impression the authors only analyzed TE genes for expression changes rather than all TEs, which may explain the low number of detected changes for TEs. I would also ask the authors to complement the metagene plots with boxplots to allow a statistical assessment of possible changes of DNA methylation.**

We would like to thank the editor for these important comments.

We are now providing detailed transcriptomic analyses of *pmd*, *pp7l-2* single and *mail1-1 pp7l-2* double mutants (Fig 6 and S5 Fig). We are also precisely describing the identification of loci defined as reproducibly misregulated in *main-3* and *ddc main-3* (Fig 2 and S2 Fig) as well as in *main-2* and *mail1-1* mutants (Fig 3 and S3 Fig). The lists of misregulated loci in *main-2* and *mail1-1* are now based on three independent RNA-seq experiments (Exp1, Exp2 and Exp3, S3 Table). As we only consider the loci that are reproducibly misregulated in all the RNA-seq experiments, these lists are slightly different than the original lists described in the first version of the manuscript. This is precisely explained in the text, as well as in S3 Fig and S2-S3 Tables.

In this study, all TEs were analyzed using TAIR10 annotation: https://www-arabidopsis-org.insb.bib.cnrs.fr/download_files/Genes/TAIR10_genome_release/TAIR10_transposable_elements/TAIR10_Transposable_Elements.txt.

Finally, as suggested by the editors and reviewers, we have performed additional DNA methylation analyses, including box plots to statistically assess DNA methylation level in *main-2* (Fig 4 and S4 Fig).

Based on the reviews, we will not be able to accept this version of the manuscript, but we would be willing to review again a much-revised version. We cannot, of course, promise publication at that time.

Should you decide to revise the manuscript for further consideration here, your revisions should address the specific points made by each reviewer. We will also require a detailed list of your responses to the review comments and a description of the changes you have made in the manuscript.

If you decide to revise the manuscript for further consideration at PLOS Genetics, please aim to resubmit within the next 60 days, unless it will take extra time to address the concerns of the reviewers, in which case we would appreciate an expected resubmission date by email to [plosgenetics@plos.org](mailto:plosgenetics@plos.org).

If present, accompanying reviewer attachments are included with this email; please notify the journal office if any appear to be missing. They will also be available for download from the link below. You can use this link to log into the system when you are ready to submit a revised version, having first consulted our [Submission Checklist](http://journals.plos.org/plosgenetics/s/submit-now#loc-submission-checklist).

To enhance the reproducibility of your results, we recommend that you deposit your laboratory protocols in [protocols.io](http://protocols.io), where a protocol can be assigned its own identifier (DOI) such that it can be cited independently in the future. For instructions see our [guidelines](http://journals.plos.org/plosgenetics/s/submission-guidelines#loc-materials-and-methods).

Please be aware that our [data availability policy](http://journals.plos.org/plosgenetics/s/data-availability) requires that all numerical data underlying graphs or summary statistics are included with the submission, and you will need to provide this upon resubmission if not already present. In addition, we do not permit the inclusion of phrases such as "data not shown" or "unpublished results" in manuscripts. All points should be backed up by data provided with the submission.

While revising your submission, please upload your figure files to the [Preflight Analysis and Conversion Engine](http://pace.apexcovantage.com/) (PACE) digital diagnostic tool.  PACE helps ensure that figures meet PLOS requirements. To use PACE, you must first register as a user. Then, login and navigate to the UPLOAD tab, where you will find detailed instructions on how to use the tool. If you encounter any issues or have any questions when using PACE, please email us at [figures@plos.org](mailto:figures@plos.org).

PLOS has incorporated [Similarity Check](http://www.crossref.org/crosscheck.html), powered by iThenticate, into its journal-wide submission system in order to screen submitted content for originality before publication. Each PLOS journal undertakes screening on a proportion of submitted articles. You will be contacted if needed following the screening process.

To resubmit, use the link below and 'Revise Submission' in the 'Submissions Needing Revision' folder.


We are sorry that we cannot be more positive about your manuscript at this stage. Please do not hesitate to contact us if you have any concerns or questions.

Yours sincerely,

Claudia Köhler
Associate Editor
PLOS Genetics

Wendy Bickmore
Section Editor: Epigenetics
PLOS Genetics


Reviewer's Responses to Questions

Comments to the Authors:
Please note here if the review is uploaded as an attachment.

**Reviewer #1: In the current submission, Nicolau and colleagues combine genetic, genomic, and phylogenetic approaches to provide new insights into the roles of two plant mobile domains (PMD) proteins, MAIN and MAIL1, in regulating the expression of both transposable elements (TE) and protein coding genes. Previously, mutants in these two gene were shown to be required for TE silencing, genome stability and plant development. However, it remained unclear how these factors fit into other known transposon silencing pathways and how, mechanistically, they control gene expression and developmental processes. Here, the authors conducted an EMS-based enhancer screen in a drm1,drm2,cmt3 (ddc) background already deficient in 2 of the 3 main DNA methylation pathways and identified two new alleles of *morc6* (a previously characterized silencing factor that acts downstream of DNA methylation) and one new allele of *main*. On a global scale they were able to show that the *ddc main-3* quadruple mutant has a much stronger phenotype than the *ddc* or *main-3* mutants alone, demonstrating that these factors likely act in independent pathways that together are required to repress TEs, especially those located in pericentromeric heterochromatin. To gain mechanistic insight into how MAIN1 functions, the authors affinity purified both MAIN and the related MAIL1 proteins using complementing, epitope tagged, transgenic plant lines and identified PP7L as a co-purifying factor by Mass Spectrometry. After confirming these interactions by as series of co-IP experiments, the authors then focused on determining whether *pp7l* mutants showed similar phenotypes as observed of *main* and *mail1* mutants. Indeed, *pp7l* mutants show similar growth defects (i.e. they are developmentally delayed) and by mining of publicly available transcriptomic data they found that although *pp7l* mutants show stronger gene expression defects, a significant subset of upregulated TEs and mis-regulated genes are shared with the *main* and *mail1* mutants. Further investigation into these common genic targets revealed an over-represented motif, suggesting this complex (either directly or indirectly) regulates gene expression via recognition of a conserved cis-regulatory element. Finally, the authors noted that while MAIN and MAIL1 associated with a PPP domain protein (*i.e.* PP7L) other PMD proteins themselves already contain PPP domains. To better understand the relationships between these domains, the authors conducted phylogenetic analyses that identified two independent case for the association and neofunctionalization of PMD-C and PP7 domains. Taken together these studies demonstrate that MAIN forms a stable complex with MAIL1 and PP7 and that all these factors influence the expression of both genes and TEs, some of which are associate with a shared cis-regulatory motif. In addition, they show that the relationship between PMD and PPP domains is conserved, suggesting this combination of motifs might represent a new protein module important for gene regulation. Although some clarifications/analyses are needed (see below), the data are well controlled, support the authors main conclusions, and advance our understanding of how the MAIN complex influences gene expression.**

**Comments**

1. **In several places the authors mention MAIN acting synergistically and redundantly with DNA methylation to silence TEs. This is confusing as written since it is not possible for MAIN to be both at any given site. Rather, MAIN is either synergistic or redundant depending on the locus. The authors should clarify this throughout.**

We would like to thank the reviewer for this comment. We have rewritten the main text, and rearranged the figures to make it clearer. We are now saying that ‘depending on the locus, the three pathways act redundantly or synergistically to repress TEs.’

**On page 6, line140-143: For the text introducing this paragraph it is hard to determine what is new data and what is referring to previously published findings. Please clarify.**

We apologize for the confusion. We are now clearly describing in the text that our *main-2* and *mail1-1* RNA-seq analyses are based on two independent experiments (Exp1 and Exp3) performed in our lab, and the reanalysis of previously published RNA-seq datasets (Exp2) from Ikeda et al. [15]. The new Figure 3, S3 Fig, S3 and S4 Tables are describing this analysis.

**For the qPCR in Fig.1C/F it is not completely clear how the data is being normalized. From the legend it seems it might be normalized to both the WT control and a house keeping gene?**

A. **Please clarify the normalization procedure and if it is double normalized please clarify why this is required as it could affect the data interpretation especially if the genes/TEs being assayed are very lowly expressed in the control plants.**

We always performed RT-qPCR analyses the same way based on the 2–∆∆Ct method. The ∆Ct is calculated using the housekeeping *RHIP1* gene. ∆∆Ct is then obtained by subtracting the wt ∆Ct from the ∆Ct of each sample. Values are eventually represented on bar chart relative to wt. The normalization procedure is now added in the Material and Methods section.

B. **To convey the robustness of the gene expression changes for these mutants and complement the data shown in Fig. 1F, please include heatmaps showing the expression of levels of the genes in the different categories shown in Fig. 1E using the RNAseq data from these mutants. Perhaps a heatmap would be a nice format to use.**

We thank reviewer #1 for this suggestion, and we are now showing heatmap analyses for all RNA-seq experiments described in the manuscript. We agree that a heatmap is a much better format to describe the different classes of upregulated TEs shown in the new figure 2. Heatmap analyses are also used in the new figure 3 and figure 6 to describe misregulated loci in *pmd* and *pp7l* single and double mutants.

2. **Is the qPCR shown in Fig 3D-F normalized differently than in Fig 1? The descriptions of the normalization in the legends differ. If they are the same, please make the descriptions similar. If they differ, please reanalyze the data in a similar manner so the data can be compared between figures.**

As mentioned in response to comment 3.A, RT-qPCR analyses are always performed the same way. We have now clarified the normalization procedure in figure legends, and we use exactly the same sentence every time.

3. **It is not clear why the authors don’t include their *main-3* mRNA seq data for protein coding genes in any of the figures. The DE genes are listed in the supplemental table, but this information is not included in Fig S2C and it is not directly compared to the previously published *main-2* data. Please include this comparison. Even if the *main-3* point mutant is weaker than the null *main-2* allele, one would still expect a similar trend.**

This analysis was an important piece of information missing in the first version of the manuscript. We are now including this comparison in figure 3.

4. **Please provide tables for the NGS data showing what was sequenced and the relevant mapping and coverage statistics.**

Table S14 is giving all the necessary information about the NGS data used in this manuscript.

5. **For their complementation assays, the authors look at the expression of several TEs in Fig. S2F, but they don’t show data for any protein coding genes. Do these lines also complement at genes?**

Yes, epitope-tagged MAIN and MAIL1 versions complement gene expression defects observed in the respective mutants. Figure 5 is now showing these complementation assays.

6. **It would be helpful to provide more information about the connections between the roles of the MAIN/MAIL1/PP7L complex and DNA methylation. MAIN has a clear connection based on the *ddc main-3* phenotype. However, it remains unclear if MAIN has two separate roles, one in connection to DNA methylation and another in connection with MAIL1 and PP7L. Perhaps addressing some of the following questions will shed light on this aspect of MAIN function:**
   a. **Are there significant changes in methylation outside of the DE genes and TEs in *main* mutants?**

Box plots analyses depicted in the new figure 4 are showing that DNA methylation is slightly, but significantly, increased at CHG and CHH sites in all TEs and pericentromeric genes. However, CHG and CHH hypermethylation is not observed at TE and genes that are misregulated in *main-2*. This suggests non-CG hypermethylation is not correlated to misregulation of TEs and genes observed in *main-2*.

b. **Is there methylation at the C’s within the CxTGxCGTTT motif in the promoters of the genes that are mis-regulated in the *main*, *mail1*, and/or *pp7l* mutants? If so, does the methylation at these specific sites change in the *main* mutant based on the BS-seq data?**

Using our BS-seq data and publicly available DNA methylome datasets (UCSC genome browser [26], we analyzed the DNA methylation level of the ‘DOWN’ motif in the promoters of the 18 downregulated genes carrying the motif. We did not detect DNA methylation in either wt or *main-2*. This is mentioned in the text.

c. **Are the DE genes and TEs in *pp7l* also enriched in pericentromeric heterochromatin? Do they overlap with any of the DE genes/TEs in the *ddc main-3* mutant?**

We only have one set of RNA-seq data (Exp3, four biological replicates) describing the DE genes and TEs in *pp7l-2* and in *mail1-1 pp7l-2* (Fig 6, S5 fig and S6 Table). These DE loci are numerous, and spread over the five chromosomes. As mentioned in the text, further transcriptomic experiments will be required to precisely define the lists of reproducibly misregulated loci in *pp7l* and *mail1-1 pp7l-2*, and thus, determine whether these loci are enriched in pericentromeric heterochromatin. However, using the lists of reproducibly misregulated loci in *main-2*, *mail1-1, main-3* and *ddc main-3*, we have confidently identified the loci that are commonly misregulated in all the mutant backgrounds used in this study (S7 and S8 Tables). In S7 and S8 tables, we are also showing that all the commonly upregulated TEs and a proportion of upregulated genes are pericentromeric.

d. **Does the *ddc pp7l* mutant phenocopy the *ddc main-3* mutant in terms of gene/TE mis-regulation?**

We agree, it will be important to determine whether *ddc pp7l-2* and *ddc main-3* share common sets of DE genes and TEs. Generating the *ddc pp7l-2* quadruple is time-consuming, therefore we could not include this analysis in the revised version of the manuscript. As mentioned in the discussion, future study will address this point.

**In the absence of additional support connecting the MAIN/MAIL1/PP7L complex to DNA methylation, the possibility that MAIN serves a dual role in several different gene regulation pathways for TE vs gene regulation should be included in the discussion.**

We are now discussing the fact that CHG and CHH methylation levels are slightly increased in *main-2*. We have also added in the discussion the possibility that MAIN is playing a dual role.

7. **The phylogenetic analyses appear to be sound. However, as I am not an expert, I defer to other reviewers should issues be raised.**

**Minor comments:**

**-) In the methods (page 14, line 353) the authors mention in vitro plant culture, but this is not mentioned in the rest of the methods. Please clarify what experiment used plant cultures.**

We are now mentioning in the materials and methods that “All the WT Col and T-DNA mutant plants were grown on soil under a 16h-light/8h-dark cycle. When experiments required to screen for GFP expression under UV light, plants carrying the *ATCOPIA28::GFP* transgene were first grown on Murashige and Skoog (MS) plates under continuous light, 10-day old plants were then screened for GFP expression under UV light, and subsequently transferred onto soil.” Furthermore, “Total RNA was extracted from aerial parts of 3-week-old seedlings grown on soil”. Finally, we have added this sentence at the end of Fig 1 and S1 Fig legends: “Screening of EMS mutant populations was done on MS plates to allow for visualization of GFP-positive individuals under UV light.”

**-) For Figure 2A, please address the following:**

**A. It is not clear from the methods that the controls were done in parallel with the MAIN and MAIL1 IPs and if they were all done in parallel, why there are only two experiments for MAIL1. Please clarify.**

We have rearranged the IPMS table, now described in Fig 5F, to make it clearer. The table is now showing that two independent experiments were carried out in this analysis.

**B. Are these the only proteins identified consistently between replicates or are these just the top hits? I was not able to access the MS data using the information provided with the submission, but regardless, such information should be mentioned, even if briefly.**

MAIN, MAIL1 and PP7L were the only three proteins reproducibly enriched in all the FLAG-MAIN and FLAG-MAIL1 IP, and depleted in WT controls across multiple replicates. We are now mentioning that in the text.

**-) In Fig 3 please add the allele information for each mutant and, as was done in the supplemental figure, please indicate in the legend the references for the mRNA-seq datasets analyzed for each of these mutants.**

Descriptions of mutant alleles have been added in the new Fig 6. We are also indicating in the main text and in S3 Fig legend that the datasets of RNA-seq Exp2 of *main-2* and *mail1-1* are based on reanalyzing RNA-seq from reference [15].

**-) In Fig S1C it would be helpful to mark the relevant interval on each of the three plots.**

A 5Mb interval was added on each graph depicted in panels S1B and S1D.

**-) In Fig S3G and Table S3, I believe the number of downregulate genes for *ddc main-3* is 87 not 88. Please fix as needed.**

We redefined the list of reproducibly misregulated loci in *ddc main-3* and realized that one locus (AT5G53210) was missing in the original list of downregulated loci (old S1 Table). This mistake is now fixed, and the new S2 Table is showing that 88 genes are reproducibly downregulated in *ddc main-3*.  **Reviewer #2: This is a review of the manuscript entitled “The plant mobile domain proteins MAIN and MAIL1 interact with the phosphatase PP7L to regulate gene expression and silence transposable elements in Arabidopsis thaliana” submitted to PLoS Genetics. In this manuscript a forward genetic screen is performed to identify factors responsible for repressing a TE-reporter transgene in an already sensitized DNA methylation-deficient background. The screen hits upon MAIN, a transposable element (TE)-derived domesticated protein that has been described before, but the molecular function of which is unknown. The strength of the manuscript is the analysis of TE activation in Figure 1 and the IP-Mass Spec analysis in Figure 2. The first two figures, along with their corresponding supplemental data, are very strong. Figure 3 and 4 are the weakness of the manuscript, as a more convincing analysis of dataset overlap needs to be done for Figure 3, and Figure 4 is highly descriptive, and ends the manuscript on a low note. Overall, it is my belief that upon revision this work could be suitable for publication in PLoS Genetics.

Major comments
1. The analysis of Figure 3A-C is not convincing to the reader. Rather than just explaining how many genes or TEs are shared between datasets, the others should perform a statistical analysis of observed compared to expected overlap based on the size of these datasets, and show p-values for how statistically enriched the overlap is. The same for the Venn diagram in Figure 1E. For example, there are so many genes and TEs in the genome, that the numbers of overlap in Figure 3A-C may simply be due to the false discovery rate (especially the very low numbers of Figure 3A). Besides this observed vs. expected analysis, to convince the readers that MAIN, MAIL1 and PP7L have similar sets of mis-expressed genes and TEs, the authors should perform a principle component analysis that shows that the expression patterns of these mutant genotypes cluster away from wild-type, and more importantly they cluster together.**

We thank reviewer #2 for this important comment. We are now showing PCA analyses and heatmaps for all the RNA-seq experiments carried out in this study. Regarding the Venn diagram overlaps, we have performed Fisher's exact tests that are confirming the significance of the overlaps (p-value <1,2.10e-14). This is mentioned in the figure legends.

**2. Figure S1F shows that crossing the TE-reporter transgene to the T-DNA insertion of MAIN gives the same effect as the point mutation from their screen. However, I am not convinced by the GFP fluorescence images nor the Western blot. The main mutant combined with ddc looks just like the starting ddc to me. This is an important point, and convincing data needs to be show that MAIN is the correct gene causing the effect identified in the screen. Perhaps GFP levels could be quantified in S1F?**

We agree that complementation test analyses are important experiments to show that it is indeed the mutation in *MAIN* that is causing GFP overexpression in *ddc #16*. This analysis is, however, complicated by the fact that *ATCOPIA28::GFP* is overexpressed only in F2 *ddc #16* x *main-2*, but not in F1 *ddc #16* x *main-2* plants, in which *drm2* and *cmt3* are heterozygote. We are now showing that GFP fluorescence is undetectable in F1 *ddc #16* x *main-2* plants (S1 Fig). Regarding the anti-GFP Western blot shown in S1F Fig, the difference of intensity among samples is most likely due to internal variation between individuals. To overcome this issue, we performed additional transcriptional analyses focusing on F1 *ddc #16* x *main-2*, and using as control F1 *ddc #16* x WT Columbia plants. We are now showing in S1G Fig that misregulation of several TEs, including the endogenous *ATCOPIA28,* can be complemented in F1 *ddc #16* x WT, but not in F1 *ddc #16* x *main-2*. Therefore, we can conclude that *MAIN* is the mutated gene causing TE silencing defect in *ddc#16*.

**3. I’m amazed that MAIN and MAIL1 coIP with PP7L, which just happens to be very close to the MAIL3 gene, in which two similar proteins to MAIN/MAIL1 and PP7L have been fused together. Please add discussion of this coincidence, and how it could have come to be that two proteins that work together could become fused.**

We agree that this phenomenon is fascinating. We are now discussing the concept of Rosetta stone protein, which has described that interacting proteins are sometimes fused together as protein domains in another protein.

**4. The end of the Results section becomes highly descriptive in regards to the evolutionary analysis. My suggestion is to either perform an experiment (bioinformatic or wet-bench) in this section, or reduce this analysis / description.**

We have reduced this part of the result section.  **Minor comments, but still should be fixed
1. The Introduction goes into background research on MORC and MOM1 proteins that is not necessary for the reader, and I feel that the purpose of mentioning them is to self-reference the authors’ previous work. Extraneous information and references should be removed.**

We have removed unnecessary references in this introduction, keeping only the original papers describing the MORC1/6 complex. We believe it is important to introduce MORC6 considering that we are describing two new *morc6* mutant alleles in S1 Fig.

**2. Figures S1-S3 have a lot of good and important information in them. I suggest moving the essential pieces of data out of the Supplemental section and into the main figures.**

We would like to thank reviewer #2 for this very constructive comment. Main and supplementary figures have been rearranged accordingly. Please refer to the paragraph describing figure rearrangements at the beginning of this document.

**3. More raw data should be added to Figure 2A (the IP-MS table). For example: % of the detected protein covered by the spectra, p-value of detection, number of unique spectra for that protein.**

Peptide counts and NSAFe5 values have been added in the table that is now appearing in Fig 5F.

**4. Figure S3A-C should be in the main figures. For Figure3B-C please show comparable data for MAIN and MAIL1, and see major point #1 above for a principle component analysis that could convince the reader that these mutants have similar expression patterns.**

As mentioned above, figures have been rearranged. We are now describing in Fig 3/S3 Fig and Fig 6/S5 Fig a new set of RNA-seq (experiment 3), with PCA, heatmaps, and statistical analyses of the overlaps between the DE genes and TEs in the different mutants.  **Reviewer #3: The manuscript of Nicolau and colleagues investigates the function of two Arabidopsis plant mobile domain (PMD) genes MAIN and MAIL1 in the regulation of transcription. In a forward genetic screen, the authors identify a point mutation in MAIN leading to the reactivation of a silent GFP reporter. RNA-sequencing shows that loss of MAIN leads to derepression of transposable elements (TEs) in redundant and synergistic manners to DNA methylation. Immunoprecipitation followed by mass spectrometry (IP-MS) shows that MAIN and its homolog MAIL1 are in a protein complex with the putative phosphatase PP7L. Comparison between different publically available gene expression datasets defines a common set of TEs and protein-coding genes mis-regulated in main, mail1 and pp7l mutants. Motif analyses identify a putative cis regulatory element (CRE) amongst mis-regulated genes and phylogenetic work suggests a neo-association process between PMD and PP7 domains.

The role of MAIN and MAIL1 in TE silencing is of general interest for the plant epigenetic community and the identification of PP7L as part of the MAIN/MAIL protein complex is the novelty of this work. The originality and hard work involved in performing a genetic screen has to be noted. However, many of the manuscript findings have already been published (Ikeda et al., Nature Communication 2017; DOI: 10.1038/ncomms15122). This encompasses the identification of MAIN and MAIL1 as transcriptional repressors of TEs, independently of DNA methylation. Unfortunately, the authors do not explore in more details the role of PP7L in gene expression and its interplay with MAIN/MAIL1, which are the major discoveries of this manuscript. The analysis of high-order mutants between main/mail and pp7l mutants would shed light on how these partners function. Also, there are multiple weaknesses in the methodology that need to be improved before publishing. With additional data deciphering the function of PP7L in transcriptional regulation, this work could be considered for publication in Plos Genetics.
In summary, the research and approaches are interesting but further experiments and analyses are required. I therefore positively encourage the authors to do revisions and resubmit this manuscript.


Major reviews and comments:

In general:
• Explanations in main text are often brief and sparse, thus making it difficult to understand the manuscript. Examples: genetic complementation and crosses, co-immunoprecipitations.**

We apologize for lack of clarity. We have now elaborated more detailed sentences describing genetic complementation, crosses and co-immnunoprecipitations experiments.  **Genetic complementation Fig S1F:
• Cross of ddc#16 with main-2: In F2, there is no information on the genotype of the MAIN gene. Thus complementation cannot be assessed. I believe that the authors forgot to mention that all the selected GFP positive plants were either homozygous for main-2 or main-3, which solves this issue.**

Indeed, information regarding the *MAIN* genotype was missing. This is now corrected. Among the selected F2 plants, most of them (7 out of 10) carried both the EMS and T-DNA mutant alleles. The three other plants were homozygote for the EMS allele. *main-2* homozygote plants were not selected most likely because of their strong chlorotic phenotype, which is therefore considered as ‘false positive phenotype’ for GFP expression. It is therefore difficult to screen under UV light for GFP-expressing individuals among a population of mutant plants showing a strong chlorosis phenotype (please see also below).

**• Is ATCOPIA::GFP reactivated in main-2 single mutant? Are F1 plants GFP positive? Homozygosity of CMT does not seem to be required for GFP reactivation in F2.**

This is a good question difficult to test because of the strong developmental phenotype of *main-2* null mutant plants. These plants show chlorosis and do not grow well on MS plates (see ref [16]). Therefore, during the screening of F2 *ddc #16* x *main-2* plants under UV light, the *main-2* homozygote plants were probably not selected because of their chlorotic phenotype, which was considered as ‘false positive phenotype’ for GFP expression. That is also probably why all the selected GFP positive F2 *ddc #16* x *main-2* plants depicted in S1F fig were either *main-3/main-2* or *main-3/main-3*.

F1 *ddc #16* x *main-2* plants are GFP negative, which is consistent with the fact that GFP overexpression requires both *main* and *drm2* mutations. However, as described now in S1G Fig, ‘more conventional’ complementation test analyses show that F1 *ddc #16* x *main-2* plants show TE upregulation (no complementation), while F1 *ddc #16* x WT Columbia plants show complementation of TE silencing defects. Therefore, we can conclude that *MAIN* is the mutated gene causing TE silencing defect in *ddc #16*.

**• Complementation can also be tested by transforming ddc#16 with the available flag-tagged MAIN construct.**

We agree that transforming *ddc #16* with the FLAG-MAIN construct would be a nice alternative to show complementation of *ATCOPIA28::GFP* and TE upregulation. Unfortunately, the *FLAG-MAIN* and *ATCOPIA28::GFP* transgenes are linked to the same selection marker (bar gene) conferring resistance to basta. It is therefore not possible to supertransform *ddc #16* with the *FLAG-MAIN* transgene.  **RNA-seq ddc main-3:
• This genome-wide information could be used to illustrate in more details the synergistic or redundant relationship between MAIN and DRM2/CMT3 of all individual up-regulated TEs. Figure 1D could for example be presented as heat map. For example, are the TEs up-regulated in the double mutant already partially reactivated in the single mutants?**

We thank reviewer #3 for this constructive comment that was also suggested by reviewers #1 and #2. All the RNA-seq data are now illustrated using heatmaps. Based on our RNA-seq threshold (log2≥2, or log2≤-2 ; p-adj< 0.01), Class IV TEs are only strongly reactivated in the *ddc main-3* mutant (new Fig 2).

**• What are the specific contributions of CHG and CHH methylation to TE reactivation in addition to loss of MAIN? The non-requirement of CMT3 homozygosity for GFP reactivation in F2 plants (Figure S1F) suggests that CHH, but not CHG methylation is the major silencing pathway redundant with MAIN. Looking at TE expression by RT-qPCR and GFP reactivation in double mutants, such as drm2/main-3 or cmt3/main-3, segregating from the complementation cross would be informative.**

We are now providing transcriptional profiling of several TEs in *drm1 drm2 main-3* and *cmt3 main-3* to show complex genetic interactions between the MAIN, DRM2 and CMT3 pathways. Depending on the locus, the three proteins act redundantly or synergistically to silence the TE (new Fig 2).

**• Are there protein-coding genes changing expression in these genotypes? Are these genes overlapping with the analysis performed in figure 3 with the publically available main-2 RNA-seq data?**

The new Fig 3 and S3 Fig are now showing a comparative analysis of DE genes in *main-2, mail1-1* and *main-3*. By integrating our last set of RNA-seq experiments (Exp3), this important analysis has allowed us to precisely define the loci that are commonly misregulated in these three mutant backgrounds (S5 Table).

**• The growth conditions (soil or plates) of the 3 weeks old seedlings remain unclear.**

All the transcript profiling analyses (RNA-seq and RTqPCR) were performed using 3-week-old plant grown on soil. We are clarifying this in the material and method section. We are also clarifying that plants were first grown on MS plates only if GFP screening under UV light was required for the experiment. Following the screening, 10-day-old selected plants were transferred onto soil for further analyses. This is mentioned in materials and methods.

**• The link to the deposition of the data is not valid.**

All the information and links to access raw data should be valid by now.  **Interactions MAIN/MAIL1/PP7L:
• Protein ladder sizes are missing on western blots in figure 2B-D. It would be important to add the images of the entire blots with all the protein sizes in the supplements.**

Protein ladder sizes have been added on all Western blots now described in this study.

**• The full list of identified proteins is missing. Were there additional interesting interaction partners identified with MAIN and MAIL?**

As mentioned above, MAIN, MAIL1 and PP7L were the only three proteins reproducibly enriched in all the FLAG-MAIN and FLAG-MAIL1 IP, and depleted in all WT controls across multiple replicates. We are now mentioning this in the text.

**• From the presented data, it cannot be ruled out that an additional protein is acting as bridge between MAIN/MAIL and PP7L. IP-MS of flag-tagged PP7L would strengthen the proposed protein complex. Also, has a bzip transcription factor been identified? In the discussion, the authors suggest that the MAIN/MAIL/PP7L complex recruit a transcription factor that could recognize the identified CRE motif**

We agree that from the presented data, we cannot exclude that an additional protein is required for the MAIN/MAIL1/PP7L interaction. Also, we cannot exclude that PP7L interacts with another partner independently of MAIN and MAIL1. This is now mentioned in the discussion. We did not identify a bzip TF as MAIN or MAIL1 interactor. To avoid confusion, we have removed this part from the discussion.

**• The data submitted to the MassiVE database cannot be accessed.**

We apologize for this inconvenience. This problem is not fixed.  **Transcriptional profiling of main-2, mail1-1 and pp7l-2:
• The plant growth stages and conditions between the re-analysed RNA-seq experiments are very different thus not providing ideal datasets to compare transcriptional responses between these genotypes. Main-2 and mail1-1 mutants were grown 18 days old on soil while the pp7l mutant was grown for 4 days on sterile plates supplemented with 1% sucrose.**

We agree that this analysis was not ideal. We are now providing in the figure 6 a new comparative transcriptomic analysis (RNA-seq Exp3, 4 biological replicates) using *main-2*, *mail1-1, pp7l-2* single and *mail1-1 pp7l-2* double mutant plants that were grown side-by-side.

**• Validation RT-qPCRs were performed on 3 weeks old seedlings (grown on plates or on soil?) however the mail1-1 mutant is missing.**

As mentioned above, all the transcript profiling analyses (RNA-seq and RTqPCR) were performed using 3-week-old plant grown on soil. This is clarified in materials and methods. New RT-qPCR analyses depicted in panels Fig 6H-J are including *mail1-1* as well as *main-2, pp7l-2* single and *mail1-1 pp7l-2* double mutant, using the new set of loci identified as commonly misregulated in *main-2*, *mail1-1, pp7l-2* single and *mail1-1 pp7l-2* double mutant plants described in S3, S6 and S7 Tables.

**• To understand the functional relationship between MAIN/MAIL and PP7L, it would be important to investigate transcriptional responses in double and triple mutants. This would reveal if PP7L functions exclusively with the MAIN/MAIL pathway or if PP7L has an additional, MAIN/MAIL-independent role in transcription.**

We agree that investigating the transcriptional responses of double and triple mutant is important. We are now showing transcriptomic analyses of *mail1-1 pp7l-2* double mutant plants (Fig 6). Future study will investigate the transcriptional responses in *main-2 mail1-1 pp7l-2* triple mutant plants. We are mentioning this point in the discussion.

**Motif analysis:
• For the identification of specific CRE motifs, have the authors also tested if randomly selected genes do not identify such motifs? Similarly, do genes only up-/down-regulated in single mutants have such motifs?**

Testing if randomly selected genes carry the ‘DOWN’ DNA motif is indeed an important control. We did perform this random test ten times, and found that, on average, only 8.40 % of randomly selected genes carry the ‘DOWN’ motif in their promoter (S9 Table). As mentioned in the new version of the manuscript: “We could not detect any enrichment of a DNA motif among any lists of upregulated genes. Likewise, we could not identify a DNA motif significantly enriched in the lists of downregulated genes from the RNA-seq Exp3. However, we identified a discrete DNA motif (hereafter called ‘DOWN’ motif) that was enriched in the promoters of genes that were reproducibly downregulated in *main-3*, *ddc main-3*, *main-2* and *mail1-1* mutants (S5C Fig). The ‘DOWN’ motif was further enriched and refined in the promoters of genes commonly downregulated in all the mutant backgrounds analyzed in this study: eighteen genes out of nineteen, 95% of enrichment (S5C Fig, S8 and S9 Tables).”

**• Is the CRE motif identified in up-regulated genes also found upstream of up-regulated TEs? At the genome-wide scale, how many genes do possess such motives in their promoters?**

In the first version of the manuscript, the ‘UP’ DNA motif was already not significant enriched in the list of commonly upregulated genes. Using the new lists of upregulated loci (which include analyses using the new RNA-seq datasets, Exp3), we could not detect any enrichment of a DNA motif among any lists of upregulated genes. We are therefore not describing this motif in the revised version. Nevertheless, as suggested by reviewer #3, we did perform this analysis. We did not find an enrichment of the ‘UP’ DNA motif upstream of upregulated TEs.

At the genome-wide scale, 3059 genes (11%, S5C Fig) carry the ‘DOWN’ motif in their promoter. As mentioned in the text, this percentage is in the range of genomic distribution of several DNA motifs recognized by bZIP TFs (S10 Table). Moreover, it is comparable to the percentage obtained using the random tests described above (8.40%, S9 Table).

**• Are MAIN, MAIL or PP7L binding the identified motifs? This could for example be tested by chromatin immunoprecipitation experiments in vivo or in vitro using EMSA or SELEX.**

This is an important question that we have been trying very hard to answer. At the moment, we do not have solid evidence showing that MAIN, MAIL1 or PP7L bind the DNA motif. We also do not know whether these proteins bind chromatin alone or as a complex. ChIP-seq, DAP-seq, EMSA, SELEX are definitely appropriate experiments to address this question. However, these experiments are time-consuming, and often require consequent troubleshooting. Therefore, it was not technically possible to address this point during the revision process, and further studies will be required to answer this important question. As a consequence, we have decided to described the ‘DOWN’ motif in new S5 Fig instead of new Fig 6.

**DNA methylation and chromatin compaction:
• To further show that loss of MAIN does not impact DNA methylation, DNA methylation levels should be plotted for all protein-coding genes, all TEs, and more specifically for sites that lost CHH or CHG methylation in drm2 and cmt3 mutants respectively.**

We are now showing additional DNA methylation analyses in the new figure 4. Box plot analyses are showing that DNA methylation at CHG and CHH sites is slightly but significantly increased at TEs and pericentromeric genes in *main-2* (Fig 4). Same observations are made at *cmt3* hypo CHG DMRs and *drm1 drm2* hypo CHH DMRs (S4 Fig). However, as previously observed, DNA methylation is unchanged at TEs and genes that are misregulated in *main-2*. This suggests non-CG hypermethylation is not correlated to the misregulation of TEs and genes observed in *main-2*.

**• Have the authors looked if there were subtle changes? BS-seq data allows calling differentially methylated regions (DMRs). Are there any DMRs in the main-2 mutant?**

We did perform DRM calling analysis in *main-2*, but we could only identify a few DRMs. None of them were located in or near the loci identified as misregulated in *main-2*.

**• Main and mail1 mutants were shown to display chromocenters decondensation. Is this also the case in pp7l-2? This would support a direct role for PP7L in chromatin compaction and strengthen the relationship between MAIN/MAIL1 and PP7L.**

Actually, in a previous study, it was shown that *main-2* and *mail1-1* nuclei do not display decondensed chromocenter based on DAPI-staining of DNA or H3K9me2 immunostaining [15]. We performed the same analyses using *pp7l-2* nuclei, and we obtained similar result. In this same previous study [15], the author then showed that, despite the lack of chromocenter decondensation, the 106B pericentromeric DNA repeats were more diffused in *main-2* and *mail1-1* nuclei in comparison to WT nuclei. Therefore, they conclude that heterochromatin was decondensed in *main-2* and *mail1-1*. Based on our H3K9me2 result, we can only conclude that PP7L is not required for heterochromatin condensation. Further studies will be required to test if pericentromeric repeats, such as the 106B or the 180bp repeats, are also decondensed in *pp7l-2*. This aspect has been discussed in the manuscript.

**Co-evolution analysis:
• The resolution of figure 4 is low and cannot be read.**

We apologize for the inconvenience. Nevertheless, we believe that high resolution files can be downloaded from the combined file generated during the uploading process.
