# Supplementary material for: The plant mobile domain proteins MAIN and MAIL1 interact with the phosphatase PP7L to regulate gene expression and silence transposable elements in Arabidopsis thaliana
Source: PLoS Genet. 2020 Apr 14;16(4):e1008324. doi: 10.1371/journal.pgen.1008324 (PMC7156037; doi:10.1371/journal.pgen.1008324)
Supplement: Attachment — Submitted filename: main mail1 revision review.docx [file pgen.1008324.s022.docx]

In their revised work, Nicolau *et. al* addressed my previous comments and included additional data to support their claims regarding the roles of MAIN, MAIL1, and PP7L in gene and transposon regulation. With the addition of the new data/data visualization, I have a few additional comments (see below) that would require modifications to the text and possibly the conclusions, but would not require new experiments. The main focus of these modifications would be to help clarify the connections between MAIN, MAIL1, and PP7L and DNA methylation-regulated gene/TE silencing, which is a major conclusion of this work (as highlighted in the title). Such clarification along with the very nice biochemical data showing that MAIN, MAIL1, and PP7L all interact to form a previously undescribed protein complex and the genetic data revealing that mutations in these genes result in similar developmental phenotypes will significantly advance the fields’ understanding of how these factors act to regulate gene and TE expression.

Major comments:

1. Ln 148-150, the authors state that “Class I TEs are upregulated in both ddc and main-3 mutants, and combining these mutations in ddcmain-3 lead to synergistic effects” and referenced Fig 2E. However, in the heatmap the difference in color is not very striking and the scale is only from -2 to 2. Thus, its not clear that this interaction is very synergistic. Perhaps a boxplot, with statistical tests, would show this more clearly?
2. For much of the RNA seq data presented, there are clear batch effects. Such variation commonly occurs in RNA seq experiments and thus packages, like DESeq2 (used in this study) include processing steps to account for batch effects. Instead of presenting the DE genes independently and then showing the overlaps (which unnecessarily complicates the text and figures) the authors should reprocess their data with a batch correction and update the associated figures/conclusions accordingly.
3. Overall it is very hard to keep clear what RNA seq data sets are being included for the various analyses, for example in Fig 6F the number of genes is different from those showing in the Fig 6B heatmap. This is the case for many of the experiments. Perhaps analyzing the data with batch correction will allow the data to be presented in a less parsed manner. If not, the authors should make it more clear in the main text and/or figure legends exactly what data is being presented.
4. Another point regarding the RNA seq is that between experiments the conclusions are not as clear as they appear to be when presented in isolation.
   a. For example, in the ddc, main-3, ddcmain-3 data set it is the upregulated genes and TEs that overlap the best between the 3 mutants (Fig 2) suggesting the act to repress the expression of a common set of genes. But for the main-2, main-3, mail1-1 it is the downregulated genes that are in common (Fig. 3) and the upregulated gene are not fully discussed. It should be stated explicitly whether the upregulated TEs and genes (at least in the mail1-1 and main-2 null mutants) from Fig. 3 overlap with the reactivated genes and TEs in the ddc/ddcmain-3 datasets (Fig. 2). If there is no, or limited overlap, then that raises the question of whether the roles of both main and mail1 are connected to the reactivation of TE and genes in association with DNA methylation as suggested by the authors.
   b. As another example, in Fig 6 the main-2 and mail1-1 mutants seem to overlap very well for both up and downregulated genes. Again, here there is no clear statement about how well the genes in this experiment overlap with genes misregulated in the ddcmain-3 mutant.
5. As a final point for the RNAseq, the author decided to take a very reductionist approach to reconcile differences in their RNA seq experiments, which resulted in their focusing in on relatively small subsets of genes. In the most extreme example, they focus on 19 genes downregulated by in all genotypes tested (S5C Fig.) that happens to have an enriched motif. However, this excludes much of the data, including data from the most consistent mRNA-seq data (S5 Fig.) that identified many more DE genes than the earlier data sets. As such, the authors might want to expand their hypothesis beyond regulation of such a restricted set of genes. Also, if these genes are downregulated, then what is the connection to DNA methylation?
6. Ln 158-159, the authors state “Remarkably, the genes that were upregulation in the three mutants were significantly enriched in pericentromeric regions of chromosomes, where constitutive heterochromatin resides (S3G Fig.).” However, this does not appear specific to this subset of genes from the figure. Please either show the genes specifically upregulated in ddc or main-3 single mutant are not similarly enriched or revise the sentence to reflect that genes regulated by all of these mutants, either alone or together, are enriched in pericentromeric heterochromatin.
7. Ln 187-190 the authors state “Conversely, the pmd mutant samples did not cluster together based on their genetic background, but rather based on the RNA-seq experiment (S3B Fig). This suggests that main-2 and mail1-1 perceive their environment in a similar way, leading to the misregulation of similar sets of loci in each independent RNA-seq experiment. Furthermore, this is consistent with the hypothesis that MAIN and MAIL1 act in the same pathway.” The last two sentences are an over-interpretation of the RNA-seq data and should be removed.
8. It is not clear to me how the inclusion of an arbitrary discussion of bZIP TFs strengthens this work. Please either clarify why this is relevant or remove it from the manuscript.
9. Please provide a figure reference or new data analysis to support the text in ln 228-229 “Moreover, this subtle non-CG hypermethylation does not correlate with the changes in gene and TE expression observed in main-2.”
10. Please add to the methods how the heatmaps, metaplots, and boxplots were generated as well as how DMRs were identified.

Minor comments:

1. In the figures and NGS supplemental table, please make clear the difference between “WT” and “Col”. I assume the “WT” is Col with the GFP reporter and “Col” is non-transgenic Col plants, but this is not explicitly stated.
2. For the mapping interval, the authors indicated at 5 MB scale, but what I meant from my previous comment was for a demarcation of the genomic region identified from the mapping (i.e the interval to look for the causal mutation). Please indicate this region in the supplemental figure.
3. Please indicate how the main-3 single mutant was generated. Presumably this was via backcrossing, but it is not included in the methods.
4. In several places the authors refer to “two pmd null mutants”. I assume this is in reference to main-2 and mail1-1. If so, please state this explicitly instead of introducing a new term. If the desire was to remind the readers that these two factors have pmd domains, the mutants can be included as a parenthetical.
5. Ln 185-186 the authors state that “all the WT samples tend to cluster together”. However these don’t cluster well. Rather, they cluster away from the experimental samples. Please reword for accuracy.
6. If any insights are available, please add to the discussion what the relationship could be between the chloroplast/stress role of PPL7L and its role in gene regulation. Is there an enrichment chloroplast related or stress genes from the RNAseq?
7. For S2G are DNA methylated genes including body methylated genes or just genes with non-CG methylation?
