# Supplementary material for: The plant mobile domain proteins MAIN and MAIL1 interact with the phosphatase PP7L to regulate gene expression and silence transposable elements in Arabidopsis thaliana
Source: PLoS Genet. 2020 Apr 14;16(4):e1008324. doi: 10.1371/journal.pgen.1008324 (PMC7156037; doi:10.1371/journal.pgen.1008324)
Supplement: Attachment — Submitted filename: Nicolau et al_2nd_Revision_Response_to_reviewers_v4.docx [file pgen.1008324.s023.docx]

GENOME AND PLANT DEVELOPMENT LABORATORY – LGDP

NATIONAL SCIENTIFIC RESEARCH CENTER – CNRS

UNIVERSITY OF PERPIGNAN – UPVD

January 17^th^, 2020

Dear Claudia Köhler, Wendy Bickmore and PLOS Genetics editors,

Thank you very much for still considering our manuscript entitled ‘**'The plant mobile domain proteins MAIN and MAIL1 interact with the phosphatase PP7L to regulate gene expression and silence transposable elements in Arabidopsis thaliana'** for publication in PLOS Genetics.

We have now revised our manuscript, addressing all the reviewers and editors’ comments. **The main comment was to reanalyze RNA-seq data using a batch correction step, which is what we did. Based on these new analyses, we have generated new lists of DE genes and TEs, and modified all the appropriate panels that are now based on these new datasets. This second revision of our manuscript is still composed of eight main figures and six supporting figures. There are now eleven supporting tables. The main conclusions of the manuscript are unchanged.**

In this new version, figures 1, 5, 7 and 8 remain unchanged. Figure 2 (panels B, C, D and E), figure 3 (all panels), figure 4 (panels F, G, H) and figure 6 (panels B, C and E-G) have been modified. Figures S4 and S6 are unchanged. In S1B and D Fig, mapping intervals are depicted with gray shaded rectangles. In S2 Fig, panels A, C, E and F are new, while panels B, D and G are same as before. In S3 Fig, panel A is new, panels B-D are same as before, panel E described a new analysis comparing DE genes and TEs in *main-2*, *mail1-1, ddc* and *ddcmain-3* following reviewer comment. In S5 Fig, panels A and E have been modified, and panels B, C and D are new. All the statistical analyses have been repeated using the new datasets.

Based on these new RNA-seq analyses using batch correction, we believe that the manuscript has been strongly improved.

Thank you very much for considering this new revised version of our manuscript. We hope you will appreciate all the modifications we have made, and that the manuscript is now acceptable for publication in PLOS Genetics.

Sincerely yours,

Guillaume Moissiard

LGDP UMR5096 CNRS/UPVD

Bâtiment T

58, Avenue P. Alduy - 66860 Perpignan FRANCE

Tel : +33(0) 4 68 08 67 90

[guillaume.moissiard@univ-perp.fr](mailto:guillaume.moissiard@univ-perp.fr)

http://lgdp.univ-perp.fr/index.php?page=pontvianne-moissiard-team

**Responses to Reviewers comments**

Dear Dr Moissiard,

Thank you very much for submitting your Research Article entitled 'The plant mobile domain proteins MAIN and MAIL1 interact with the phosphatase PP7L to regulate gene expression and silence transposable elements in Arabidopsis thaliana.' to PLOS Genetics. Your manuscript was fully evaluated at the editorial level and by two independent peer reviewers. The reviewers acknowledged that the revisions strongly improved the manuscript, but one of the reviewers identified several aspects that should be improved. I agree with the reviewer that the transcriptome analyses requires revisions; the highly stringent cutoffs applied together with the fact that several experiments were independently analyzed and only overlaps further considered, limits the number of identified loci, which may impact on the conclusions. Based on the reviews, we will not be able to accept this version of the manuscript, but we would be willing to review again a revised version addressing those concerns.
Your revisions should address the specific points made by the reviewer. We will also require a detailed list of your responses to the review comments and a description of the changes you have made in the manuscript.

We would like to thank reviewer #1 for the thorough review of our manuscript. We believe that his major comment suggesting to reanalyze RNA-seq data using batch correction step was very appropriate. We have now reanalyzed all the RNA-seq data for *main-2*, *mail1-1*, *ddc*, *main-3* and *ddc main-3.* We believe that using batch correction has improved our manuscript, and has made it clearer. Importantly, the main conclusions of the manuscript are unchanged.

Below are our point-by-point responses. **reviewers’ comments are in** **bold type**, and our responses are in regular type, blue color.

**Reviewer #1:**

**In their revised work, Nicolau *et. al* addressed my previous comments and included additional data to support their claims regarding the roles of MAIN, MAIL1, and PP7L in gene and transposon regulation. With the addition of the new data/data visualization, I have a few additional comments (see below) that would require modifications to the text and possibly the conclusions, but would not require new experiments. The main focus of these modifications would be to help clarify the connections between MAIN, MAIL1, and PP7L and DNA methylation-regulated gene/TE silencing, which is a major conclusion of this work (as highlighted in the title). Such clarification along with the very nice biochemical data showing that MAIN, MAIL1, and PP7L all interact to form a previously undescribed protein complex and the genetic data revealing that mutations in these genes result in similar developmental phenotypes will significantly advance the fields’ understanding of how these factors act to regulate gene and TE expression.**

**Major comments:**

1. **Ln 148-150, the authors state that “Class I TEs are upregulated in both ddc and main-3 mutants, and combining these mutations in ddcmain-3 lead to synergistic effects” and referenced Fig 2E. However, in the heatmap the difference in color is not very striking and the scale is only from -2 to 2. Thus, its not clear that this interaction is very synergistic. Perhaps a boxplot, with statistical tests, would show this more clearly?**

We thank the reviewer for this very important comment. As suggested, we performed a boxplot analysis using the new dataset of class I TEs (after batch correction). Although we could see a slight synergistic effect in *ddcmain-3* in comparison to *ddc* or *main-3*, this effect was not statistically significant. Therefore, we have decided to remove this statement from the main text.

2. **For much of the RNA seq data presented, there are clear batch effects. Such variation commonly occurs in RNA seq experiments and thus packages, like DESeq2 (used in this study) include processing steps to account for batch effects. Instead of presenting the DE genes independently and then showing the overlaps (which unnecessarily complicates the text and figures) the authors should reprocess their data with a batch correction and update the associated figures/conclusions accordingly.**

We are very grateful for this comment. We have reanalyzed all the RNA-seq data described in this study using the DESeq2 batch correction. Thus, the different batches have been processed together, which makes our analyses much clearer. Based on the new lists of DE genes and TEs, we have remade figure panels accordingly (please see above for more details). The new lists of DE genes and TEs that we generated using DESeq2 batch correction step are bigger than previously. Importantly, loci that were previously defined as DE genes or TEs in the previous lists are still present in the new lists (except one locus: AT1G21310 that is no more in the list of upregulated genes in main-2). Overall, this new analysis does not change the main conclusion of the paper.

3. **Overall it is very hard to keep clear what RNA seq data sets are being included for the various analyses, for example in Fig 6F the number of genes is different from those showing in the Fig 6B heatmap. This is the case for many of the experiments. Perhaps analyzing the data with batch correction will allow the data to be presented in a less parsed manner. If not, the authors should make it more clear in the main text and/or figure legends exactly what data is being presented.**

Using the DESeq2 batch correction has strongly improved this analysis and simplified Fig 6 and supporting material. Thus, the analyses previously described in Fig6 panels G, K-M have been removed.

4. **Another point regarding the RNA seq is that between experiments the conclusions are not as clear as they appear to be when presented in isolation. `**
   a. **For example, in the ddc, main-3, ddcmain-3 data set it is the upregulated genes and TEs that overlap the best between the 3 mutants (Fig 2) suggesting the act to repress the expression of a common set of genes. But for the main-2, main-3, mail1-1 it is the downregulated genes that are in common (Fig. 3) and the upregulated gene are not fully discussed. It should be stated explicitly whether the upregulated TEs and genes (at least in the mail1-1 and main-2 null mutants) from Fig. 3 overlap with the reactivated genes and TEs in the ddc/ddcmain-3 datasets (Fig. 2). If there is no, or limited overlap, then that raises the question of whether the roles of both main and mail1 are connected to the reactivation of TE and genes in association with DNA methylation as suggested by the authors.**
   b. **As another example, in Fig 6 the main-2 and mail1-1 mutants seem to overlap very well for both up and downregulated genes. Again, here there is no clear statement about how well the genes in this experiment overlap with genes misregulated in the ddcmain-3 mutant.**

We are now showing comparative analyses of DE genes and TEs in *main-2*, *mail1-1, ddc* and *ddc main-3* (S3E Fig). From these analyses, we conclude that *main-2*, *mail1-1* and *ddc* show only an overlap for upregulated TEs and genes but no overlap for downregulated genes (same as what was already observed between *ddc, main-3* and *ddc main-3*). As expected, there is an overlap for downregulated genes between *main-2*, *mail1-1* and *ddc main-3* (because of the presence of *main-3* mutation in *ddc main-3*). We are now including paragraphs at the end of the result section ‘**MAIN and MAIL1 are required for the proper expression of a common set of genes and TEs.’** (lines 213-222) as well as at the end of discussion section **‘Genes that are commonly downregulated in *main, mail1* and *pp7l* mutants carry the ‘DOWN’ motif in their promoters** (lines 469-477)**.** These paragraphs clarify our interpretation of the results obtained in this study.

**As a final point for the RNAseq, the author decided to take a very reductionist approach to reconcile differences in their RNA seq experiments, which resulted in their focusing in on relatively small subsets of genes. In the most extreme example, they focus on 19 genes downregulated by in all genotypes tested (S5C Fig.) that happens to have an enriched motif. However, this excludes much of the data, including data from the most consistent mRNA-seq data (S5 Fig.) that identified many more DE genes than the earlier data sets. As such, the authors might want to expand their hypothesis beyond regulation of such a restricted set of genes. Also, if these genes are downregulated, then what is the connection to DNA methylation?**

As previously mentioned, we have reanalyzed all the RNA-seq data using batch correction step. This allowed to define new lists of DE genes and TEs in *main-2* and *mail1-1* (based on 3 RNA-seq batches), and in *ddc*, *main-3* and *ddc main-3* (based on 2 RNA-seq batches). Overall, these new analyses allowed us to increase the different lists of DE genes and TEs. Nevertheless, comparative analyses led to results that are similar to the results previously described. And, conclusions remain unchanged.

The *main-2*, *mail1-1, pp7l-2* and *mail1-1 pp7l-2* null mutants display strong developmental phenotype and large numbers of misregulated loci. By comparison, the hypomorphic *main-3* mutant displays a weaker developmental phenotype, and fewer DE loci. As mentioned in the text, it is likely some of the gene misregulation observed in *main-2*, *mail1-1, pp7l-2* and *mail1-1 pp7l-2* might be due to side effects of the mutations. Therefore, including the weak *main-3* mutant allele in our analysis is a good opportunity to narrow down the number of misregulated loci, and focus our analysis on ‘real’ PMD’s and PP7L’s target genes.

Besides, all the silico analyses using the different overlaps of downregulated genes defined in this study are always identifying the ‘DOWN’ motif. Therefore, we believe that the results and conclusions are suitable, keeping in mind that we are focusing on loci that are commonly misregulated in the different *pmd* and *pp7l* mutants studied here. This is the reason why we are mentioning in the discussion that **“Transcriptomic analyses revealed complex genetic interaction between MAIN, MAIL1 and PP7L; the three proteins acting either independently or together to ensure the proper expression of genes, and to perform TE silencing.”** (line 436). Thus, we are acknowledging that MAIN, MAIL1 and PP7L are not only interacting together to regulate gene expression and silencing TEs, but it is likely that they also act independently. Finally, we are discussing that MAIN/MAIL1/PP7L-mediated gene activation most likely independently of DNA methylation. There is only a cooperation between MAIN and DNA methylation pathways such as DRM2 and CMT3 to silence subsets of TEs and DNA-methylated genes.

5. **Ln 158-159, the authors state “Remarkably, the genes that were upregulation in the three mutants were significantly enriched in pericentromeric regions of chromosomes, where constitutive heterochromatin resides (S3G Fig.).” However, this does not appear specific to this subset of genes from the figure. Please either show the genes specifically upregulated in ddc or main-3 single mutant are not similarly enriched or revise the sentence to reflect that genes regulated by all of these mutants, either alone or together, are enriched in pericentromeric heterochromatin.**

In the previous version of S2 Fig, the bottom part of panel G was a graph showing the proportion of pericentromeric vs chromosome arm-located genes among the lists of up-and downregulated genes in *ddc*, *main-3* or *ddcmain-3* in comparison to all genes. A similar graph using the new lists of DE genes (with batch correction) is now depicted in panel S2F. The same conclusions apply.

6. **Ln 187-190 the authors state “Conversely, the pmd mutant samples did not cluster together based on their genetic background, but rather based on the RNA-seq experiment (S3B Fig). This suggests that main-2 and mail1-1 perceive their environment in a similar way, leading to the misregulation of similar sets of loci in each independent RNA-seq experiment. Furthermore, this is consistent with the hypothesis that MAIN and MAIL1 act in the same pathway.” The last two sentences are an over-interpretation of the RNA-seq data and should be removed.**

These two sentences have been removed.

7. **It is not clear to me how the inclusion of an arbitrary discussion of bZIP TFs strengthens this work. Please either clarify why this is relevant or remove it from the manuscript.**

Comparison with bZIP TFs has been removed from the manuscript.

8. **Please provide a figure reference or new data analysis to support the text in ln 228-229 “Moreover, this subtle non-CG hypermethylation does not correlate with the changes in gene and TE expression observed in main-2.”**

We have reformulated this sentence, and provided appropriate figure reference.

9. **Please add to the methods how the heatmaps, metaplots, and boxplots were generated as well as how DMRs were identified.**

This information is now included in appropriate sections of the methods.

**Minor comments:**

1. **In the figures and NGS supplemental table, please make clear the difference between “WT” and “Col”. I assume the “WT” is Col with the GFP reporter and “Col” is non-transgenic Col plants, but this is not explicitly stated.**

To avoid confusion, we are now mentioning in the plant material section of material and methods as well as in S12_Table (NGS supp data) the following sentence: Col= Non-transgenic WT Columbia ecotype; WT= ATCOPIA28::GFP in WT Columbia background.

We have also provided more information regarding the lines ‘ddc’, ddc main-3’ and ‘main-3’ in the plant material section of material and methods.

2. **For the mapping interval, the authors indicated at 5 MB scale, but what I meant from my previous comment was for a demarcation of the genomic region identified from the mapping (i.e the interval to look for the causal mutation). Please indicate this region in the supplemental figure.**

The mapping intervals are depicted by shaded rectangles on the three graphs.

3. **Please indicate how the main-3 single mutant was generated. Presumably this was via backcrossing, but it is not included in the methods.**

Indeed, main-3 was generated by backcrossing *ATCOPIA28::GFP main-3 (main-3*) with *ATCOPIA28::GFP* WT (WT). This is now specified in the plant material section of material and methods.

4. **In several places the authors refer to “two pmd null mutants”. I assume this is in reference to main-2 and mail1-1. If so, please state this explicitly instead of introducing a new term. If the desire was to remind the readers that these two factors have pmd domains, the mutants can be included as a parenthetical.**

We have replaced ‘two pmd null mutants’ by main-2 and mail1-1 in the text.

5. **Ln 185-186 the authors state that “all the WT samples tend to cluster together”. However these don’t cluster well. Rather, they cluster away from the experimental samples. Please reword for accuracy.**

We are now providing a new PCA based on the reanalysis of all the RNA-seq experiments using batch correction. Based on this new PCA (S3A Fig), we are saying that “*main-2* and *mail1-1* mutant samples tend to cluster together, and away from the WT samples”.

6. **If any insights are available, please add to the discussion what the relationship could be between the chloroplast/stress role of PPL7L and its role in gene regulation. Is there an enrichment chloroplast related or stress genes from the RNAseq?**

We performed Gene Ontology term analyses using the list of DEG in pp7l-2 but we did not find any enrichment in chloroplast-related genes. Further study will be needed to determine the role of PP7L in regulation of transcription and chloroplast biology.

7. **For S2G are DNA methylated genes including body methylated genes or just genes with non-CG methylation?**

In the 1^st^ revised version, we defined as DNA-methylated genes the genes showing DNA methylation in the three cytosine contexts (not body methylated genes that are only methylated at CG sites in their transcribed region).

To improve this analysis, we are not showing the percentage of gene belonging to the chromatin states 8 and 9 (constitutive heterochromatin enriched in mC, H3K9me2, H3K27me1 and H3.1 variant) as defined in Vergara and Gutierrez, Genome Biology 2017.

As a last comment, we would like to mention that the gene AT5G09570 previously named COX19L has been renamed At12Cys-2 in accordance to Wang et al., Molecular Plant 9, 696–710, May 2016.
