# Supplementary material for: The plant mobile domain proteins MAIN and MAIL1 interact with the phosphatase PP7L to regulate gene expression and silence transposable elements in Arabidopsis thaliana
Source: PLoS Genet. 2020 Apr 14;16(4):e1008324. doi: 10.1371/journal.pgen.1008324 (PMC7156037; doi:10.1371/journal.pgen.1008324)
Supplement: Attachment — Submitted filename: Nicolau et al_3rd_Revision_Response_to_reviewers.docx [file pgen.1008324.s024.docx]

GENOME AND PLANT DEVELOPMENT LABORATORY – LGDP

NATIONAL SCIENTIFIC RESEARCH CENTER – CNRS

UNIVERSITY OF PERPIGNAN – UPVD

February 21^st^, 2020

Dear Claudia Köhler, Wendy Bickmore and PLOS Genetics editors,

Thank you very much for still considering our manuscript entitled ‘**'The plant mobile domain proteins MAIN and MAIL1 interact with the phosphatase PP7L to regulate gene expression and silence transposable elements in Arabidopsis thaliana'** for publication in PLOS Genetics.

We have revised our manuscript by addressing the two minor comments of Reviewer #1. The Western blot panels described in Fig 5B have been properly relabeled, and we added into S6 Fig the raw images corresponding to Western blot experiments described in Fig 1C and Fig 5B. In addition, we have made minor editing in material and method section to precise the procedure of plant transformation events in accordance to what is described in Fig 5. Finally, because we noticed some discrepancies between the PP7L (AT5G10900) DNA and protein sequences we used in this study in comparison to the sequences described on the TAIR website, we are now providing a S12 Table documenting the exact PP7L sequences used in our study. We believe there are two misannotations within the genomic DNA and protein sequences of PP7L described on the TAIR website, and we have informed the TAIR website curators about this issue. Nevertheless, for transparency and clarity purpose, we thought it was important to provide the exact PP7L DNA and protein sequences used in our article. We are also showing in S13 Table all the raw data related to the RT-qPCR experiments.

Thank you very much for considering this new revised version of our manuscript. We hope you will appreciate all the clarifications we have made, and that the manuscript is now acceptable for publication in PLOS Genetics.

Sincerely yours,

Guillaume Moissiard

LGDP UMR5096 CNRS/UPVD

Bâtiment T

58, Avenue P. Alduy - 66860 Perpignan FRANCE

Tel : +33(0) 4 68 08 67 90

[guillaume.moissiard@univ-perp.fr](mailto:guillaume.moissiard@univ-perp.fr)

http://lgdp.univ-perp.fr/index.php?page=pontvianne-moissiard-team

* Please note while forming your response, if your article is accepted, you may have the opportunity to make the peer review history publicly available. The record will include editor decision letters (with reviews) and your responses to reviewer comments. If eligible, we will contact you to opt in or out. *


Dear Dr Moissiard,

Thank you very much for submitting your Research Article entitled 'The plant mobile domain proteins MAIN and MAIL1 interact with the phosphatase PP7L to regulate gene expression and silence transposable elements in Arabidopsis thaliana.' to PLOS Genetics. Your manuscript was fully evaluated at the editorial level and by independent peer reviewers. One of the reviewers identified two minor issues to be addressed before the manuscript can be finally accepted.

We therefore ask you to modify the manuscript according to the review recommendations before we can consider your manuscript for acceptance.

In addition we ask that you:
1) Provide a detailed list of your responses to the review comments and a description of the changes you have made in the manuscript.
2) Upload a Striking Image with a corresponding caption to accompany your manuscript if one is available (either a new image or an existing one from within your manuscript). If this image is judged to be suitable, it may be featured on our website. Images should ideally be high resolution, eye-catching, single panel square images. For examples, please browse our [archive](http://www.plosgenetics.org/article/browse/volume). If your image is from someone other than yourself, please ensure that the artist has read and agreed to the terms and conditions of the Creative Commons Attribution License. Note: we cannot publish copyrighted images.

We hope to receive your revised manuscript within the next 30 days. If you anticipate any delay in its return, we would ask you to let us know the expected resubmission date by email to [plosgenetics@plos.org](mailto:plosgenetics@plos.org).

If present, accompanying reviewer attachments should be included with this email; please notify the journal office if any appear to be missing. They will also be available for download from the link below. You can use this link to log into the system when you are ready to submit a revised version, having first consulted our [Submission Checklist](http://journals.plos.org/plosgenetics/s/submit-now#loc-submission-checklist).

While revising your submission, please upload your figure files to the [Preflight Analysis and Conversion Engine](http://pace.apexcovantage.com/) (PACE) digital diagnostic tool. PACE helps ensure that figures meet PLOS requirements. To use PACE, you must first register as a user. Then, login and navigate to the UPLOAD tab, where you will find detailed instructions on how to use the tool. If you encounter any issues or have any questions when using PACE, please email us at [figures@plos.org](mailto:figures@plos.org).

Please be aware that our [data availability policy](http://journals.plos.org/plosgenetics/s/data-availability) requires that all numerical data underlying graphs or summary statistics are included with the submission, and you will need to provide this upon resubmission if not already present. In addition, we do not permit the inclusion of phrases such as "data not shown" or "unpublished results" in manuscripts. All points should be backed up by data provided with the submission.

PLOS has incorporated [Similarity Check](http://www.crossref.org/crosscheck.html), powered by iThenticate, into its journal-wide submission system in order to screen submitted content for originality before publication. Each PLOS journal undertakes screening on a proportion of submitted articles. You will be contacted if needed following the screening process.

To resubmit, you will need to go to the link below and 'Revise Submission' in the 'Submissions Needing Revision' folder.


Please let us know if you have any questions while making these revisions.

Yours sincerely,

Claudia Köhler
Associate Editor
PLOS Genetics

Wendy Bickmore
Section Editor: Epigenetics
PLOS Genetics

**Responses to Reviewers comments:**

We would like to thank again Reviewer #1 for the thorough review of our manuscript, and for contributing to the improvement of this article.

Below are our point-by-point responses. **Reviewers’ comments are in** **bold type**, and our responses are in regular type, blue color.

**Reviewer #1: In their revised manuscript Nicolau et al. addressed all my previous concerns and now present a much more compelling set of transcriptomics data. I have no addition major issues and the minor issues listed should not warrant additional review.

Minor Comments
1. Fig 1C, please add the raw image of the gel to the figure with the rest of the gels.**

Raw image of the Western blot described in Fig 1C is now shown in S6 Fig.

**2. Fig 5B, the gel is labeled MAIN and MAIL1-Flag, but the gels are Flag and Myc. Please relabel for clarity and also add the raw images of these gels to the figure with the rest of the gels.**

We have properly relabeled the two Western blots described in Fig 5B. Thank you for noticing that!

In addition, the raw images of these Western blots are now shown in S6 Fig.
